# Peristaltic transport of Rabinowitsch nanofluid with moving microorganisms

**DOI:** 10.1038/s41598-023-28967-5

**Published:** 2023-02-01

**Authors:** Galal M. Moatimid, Mona A. A. Mohamed, Khaled Elagamy

**Affiliations:** Department of Mathematics, Faculty of Education, Ain Shams University, Roxy, Cairo, Egypt

**Keywords:** Materials science, Mathematics and computing, Nanoscience and technology, Applied physics, Fluid dynamics, Statistical physics, thermodynamics and nonlinear dynamics

## Abstract

The key objective of the current examination is to examine a symmetrically peristaltic movement of microorganisms in a Rabinowitsch fluid (RF). The Boussinesq approximation, buoyancy-driven flow, where the density with gravity force term is taken as a linear function of heat and concentrations, is kept in mind. The flow moves with thermophoretic particle deposition in a horizontal tube with peristalsis. The heat distribution and volume concentration are revealed by temperature radiation and chemical reaction characteristics. The originality of the existing study arises from the importance of realizing the benefits or the threats that nanoparticles, microbes, and bacteria cause in the flow inside peristaltic tubes. The results are an attempt to understand what factors perform additional advantages and or reduce damages. The controlling nonlinear partial differential equations (PDEs) are made simpler by employing the long wavelength (LWL) and low-Reynolds numeral (LRN) approximations. These equations are subjected to a set of non-dimensional transformations that result in a collection of nonlinear ordinary differential equations (ODEs). By employing the Homotopy perturbation method (HPM), the configuration of equational analytical solutions is examined. Analytical and graphical descriptions are provided for the distributions of axial speed, heat, microbes, and nanoparticles under the influence of these physical characteristics. The important findings of the current work may help to comprehend the properties of several variations in numerous biological situations. It is found that the microorganisms condensation decays with the rise of all the operational parameters. This means that the development of all these factors benefits in shrinking the existence of harmful microbes, viruses, and bacteria in the human body’s peristaltic tubes, especially in the digestive system, and large and small intestines.

## Introduction

Due to its extensive mechanical and biological use, the peristaltic movement of liquids has recently gained a lot of interest. It is a form of liquid movement that develops physiologically in the human body. Several of its characteristics were seen in biological formations. Through waves of expansion and contraction, fluid was transferred from the low-pressure region to the high-pressure region during peristalsis. This phenomenon affects the biological motion of liquids in a variety of physiological systems, including the transmission of food bolus via the esophagus, the flow of urine from the kidneys to the bladder, the circulation of blood in tiny blood vessels, and chyme movement in the digestive tract. Latham^1^ was considered the first who organized an attempt at peristaltic transport flow in a pump. In circular tubes and channels, the peristaltic motion under LRN and LWL was reported^2^. Numerous researchers have been studying peristaltic transport issues with various geometries in recent years due to their practical products in the manufacturing and medical fields. A few examples of flows requiring peristaltic transport include the flow of urine from the kidney to the bladder, the development of food in the intestinal system, intrauterine liquid movement, and vasomotor of the tiny blood arteries. The development of finger pumps, roller pumps, and blood pumps were just a few industrial applications that have made use of this peristaltic transport mechanism. Investigations were made on how an endoscope and heat transmission affected the peristaltic movement of an incompressible Walters B liquid in an inclined tube^3^. It was found that the volume flow rate, the heat generation factor, and the inclination angle all had an increasing effect on the pressure gradient. When the endoscope radius grows, the pumping was at its finest. On the peristaltic movement of an MHD Walters B liquid across a permeable material in an inclined asymmetric channel, the consequences of heat transmission were investigated^4^. Since no research was done on the impact of MHD on the peristaltic flow of a Walters B liquid across a permeable material in an inclined asymmetric channel with heat transmission, the issue was novel. It has been documented that Ellis' liquid, in a symmetric tube with compliant walls, can transmit temperature and homogeneous-heterogeneous reactions for the peristaltic transport phenomena^5^. Outcomes for several Newtonian and non-Newtonian prototypes were carefully considered. The work has a large variety of products in biomedical science. Through the use of a homotopy-based approach and fractional calculus, the thin film flow of non-Newtonian pseudo-plastic liquid on a vertical wall was examined^6^. Additionally, in fractional space, the effect of numerous factors on speed was also investigated. Asymmetric compliant channels with rheological properties, improving damping tools, safety apparatus individuals, and numerous distinctive technical techniques were all considered in the analysis of the effects of thermal radiation and slips on the peristaltic flow of Sisko fluid^7^. The Adomian decomposition approach was used to study the peristaltic flow in an inclined asymmetric channel with mass and heat transmission^8^. Using the conventions of LRN and LWL approximations, the resultant equations were simplified. The movement was evaluated in a wave frame of reference traveling at the speed of the wave. In a non-uniform inclined tube, the impacts of the wall characteristics and the Cu-water nanofluid were examined^9^. In this work, the temperature and velocity slip effects were also considered together with the two-dimensional flow of a viscous nano liquid produced by peristaltic motion. Using the LWL approximation, the characteristics of peristaltic constructions were defined by the domination of viscous forces over inertial impacts. Analysis was done on the Casson fluid peristaltic transport in the existence of mass and heat transfer as well as the effects of slip conditions and wall characteristics in a non-uniform inclined tube^10^.

Non-Newtonian liquids have many uses in manufacturing and business, which has rekindled attention among academics. The removal of crude oil from petroleum products, food mixing, oscillatory flow in the bowel, and the flow of plasma, blood, nuclear fuel slurries, fluid metals, and mercury amalgams were a few examples of these applications. Elasto-viscous fluids have drawn a lot of research interest in non-Newtonian fluid models because they were vital in solving many technical issues, including the manufacture of plastic harvests as rayon and nylon, the purification of crude oil, the pulp and paper manufacturing, the textile engineering, the treatment of environmental pollution, the petroleum manufacturing, and the purification of rainwater^11^ and^12^. A behavioral analysis of the magnetically affected three-dimensional (3D) squeezing movement of nano liquids in a rotational channel was conducted^13^. Water was used as the base liquid in the channel because it contains a variety of nanoparticles, including silicon, copper, silver, gold, and platinum. For the resolution of a highly nonlinear interacting system, the HPM was used. Oldroyd liquids are a type of Maxwell fluids that have significant impacts in a variety of engineering, scientific, and manufacturing uses. Consequently, employing the Oldroyd 6-Constant model for both lifting and draining situations, a study in fractional space on Oldroyd liquids in a thin-film movement environment was described^14^. One of the non-Newtonian models that were predicated as being necessary for comprehending the complicated rheological characteristics of biological fluids was the RF. Its cubic stress prototype showed the properties of Newtonian liquids like air and water, shear-thinning or pseudoplastic liquids like blood plasma, ketchup, and syrup, and shear-thickening or dilatant liquids like sand, oobleck, and polyethylene glycol. The traditional performance of non-Newtonian liquid flows has gained importance in recent years in biological, medicinal, and manufacturing uses. The framework was developed by Rabinowitsch^15^ for examining the fundamental characteristics of non-Newtonian fluids. It investigated how convective heat transmission and varied liquid characteristics affected the peristaltic structure of RF in a small permeable channel^16^. It also explored how inclination might affect the complaint channel walls. An RF model mechanical characteristics and the influence of heat conductivity on them were investigated^17^. The flow was thought to be caused by a metachronal wave that was created by cilia continuously beating against the walls of a horizontal circular tube. Wall characteristics investigation and mathematical model for the peristaltic transport of the RF type in a non-uniform tube with coupled impacts of viscous dissipation and convective border requirements were taken into consideration^18^. In everyday applications including machinery, the human body, and medical devices, the movement of non-Newtonian liquids in tubes and pipelines is crucial. Owing to a number of factors, Singh, and Singh’s^19^ study of these phenomena using RF was not found to be sufficient, and numerous non-Newtonian fluid models have occasionally been taken into consideration by investigators. Utilizing the RF model and the LWL and LRN approximations, the issue of temperature transmission and peristaltic transport of non-Newtonian liquid was explored^20^. They found expressions for temperature, friction force, and pressure gradient. in the peristaltic stream of an RF in an inclined channel, the effects of the compliant wall and the varying fluid characteristics were investigated^21^. The liquid had varying viscosities depending on the channel thickness, and heat-dependent thermal conductivity was also considered. The main goal of this research is to investigate mixed convection flow modeling for RF physiological transport under convective settings^22^. In an inclined tube, peristaltic flow motion was considered. The RF also considered the impacts of mixed convection and convective boundary restrictions. The non-Newtonian RF was explored in the process of studying peristaltic flow in a tube^23^. Solutions for a liquid movement in the axial direction in terms of pressure gradient were found when considering the main factors in the Navier–Stokes equations. For the RF prototype with the stiffness and dynamic damping effects through the Darcy-Brinkman-Forchheimer permeable medium, the impact of heat and mass transport on particle-liquid suspension was investigated^24^. Results were carefully considered for several liquid prototypes (thinning, thickening, and viscous models). For shear thinning, it was discovered that the speed distribution improves for higher amounts of the viscous damping impact and the stiffness and rigidity factor, while the thickening nature modeling displayed contradictory behavior. To explain the effects of the Hall current and Joule heating on the peristaltic movement of blood movement, a theoretical framework was presented^25^. In the case of minor stenosis, the flow travels via a tapered artery. An external, consistent magnetic field was used to carry out the process. The RF can determine the blood structure.

Bioconvection has several uses in both biological systems and nanomaterials. This depicts a density gradient affected by bacterial motility that causes convective heat transport in a macroscopic fluid. These self-propelled motile bacteria raise the concentration of the support liquid by traveling in a certain path, which results in bio-convection. The occurrence of bioconvection in nano-liquid convection was driven by the existence of heavy microorganisms that gather on the side of light liquid. Macroscopic movement in these phenomena was caused by minute bacterial motility. The Brownian flow and thermophoresis in the nano liquid push the nanomaterials. Therefore, the movement of the bacterial flagella is unaffected by the mobility of nanomaterials. Adding microorganisms to a nano liquid increased the stability of the suspension provided^26^. The steady-state transfer boundary layer movement with a powerful object confined in a permeable environment packed with nanoparticles, including gyrotactic microorganisms, was computationally investigated^27^. They claimed that the volume, temperature, and ratio of the transfer of motile bacteria were all considerably impacted by the bio-convection variables. In both the heated and cooled sphere cases, the steady mixed convection border layer movement around a solid sphere with a constant surface heat surrounded in a permeable medium filled with a nano liquid incorporating gyrotactic microorganisms in a stream flowing vertically upwards was numerically studied^28^. A porous upright running barrier was utilized to study the bio convection brought on by the hydromagnetic movement of a novel liquid-based nano liquid including motile microorganisms and nanoparticles^29^. The MHD lamina boundary layer flow was seen to occur on a mixed convection stretchable surface with an electrically conducting water-based nano liquid containing gyrotactic microorganisms^30^. A thorough explanation of bioconvection in suspensions of oxytactic bacteria was produced^31^ for the beginning of bioconvection in a suspension of gyrotactic/oxytactic microorganisms under varied circumstances. The influence of small, powerful particles on a diluted liquid containing gyrotactic microorganisms was calculated, and the effect of bio convection on tiny solids was evaluated using the concept of successful diffusivity. Technology development and application greatly rely on how microbes behave naturally at air-fluid interfaces and contact line dynamics. The formation of centimeters-scale droplets was already studied^32^. The peristaltic movement of Carreau-Yasuda liquid around a micro-vessel containing oxytactic bacteria and nanomaterials was studied in a vertically asymmetric channel^33^. Although denitrifying bacteria display unfavorable chemotaxis to oxygen gradients, physiochemical characteristics research discovered that malignant cells might even reach healthy tissues when exposed to low oxygen levels (oxygen repellents). Therefore, it is necessary to look at the actions of oxytactic microorganisms and nanoparticles, as well as their roles in the drug-carrier system. Through the MHD flow of an incompressible nanofluid adhering to the non-Newtonian Jeffrey model, the behavior of motile microorganisms was studied^34^. Due to its many benefits, including flow control in fluidics networks, fluid pumping, thermal reactors, mixing, liquid stirring, liquid chromatography, and micro coolers, electromagnetic hydrodynamics (EMHD) was particularly significant. Based on these uses, slip effects were applied to the electromagnetic forces on the water flow containing microorganisms across a Riga plate^35^.

Numerous important phenomena were governed in nature by nonlinear PDEs. Since exact solutions are generally unreachable, numerical, experimental, and analytical solutions were considered. In these situations, a perturbation method was necessary. Engineers have frequently used a variety of perturbation techniques to address a wide range of real-world engineering issues. Even though they caused practical challenges, these approaches have significant drawbacks. They assume a little or big parameter, which implies that at least one unknown must be characterized by several slight parameters. This does not always happen since not all nonlinear equations have a small parameter. The findings of these methodologies were often accurate for tiny values of this parameter, even if such a little parameter occurred. Initial and boundary conditions may not always be required for simplified linear equations since the resulting linear equation frequently differs from the original non-linear equation in important ways. The initially related approximations were hence distant from the accurate formula. The main cause of the shortcomings of many perturbation procedures was the small parameter assumption. It seemed that most non-linear situations with no small parameters need fresh approaches. The HPM was recently developed to find the analytical solution for a differential equation. It is currently of great interest to many fields. This was because it may address several mild nonlinear difficulties at once. The original idea for HPM was put forward by the Chinese mathematician Prof. He^36^. This approach, when compared to other analytical processes, makes computing analytical results simpler and faster, and various academics have utilized it as an example in their respective fields of study. Thus, HPM-based methods for resolving nonlinear problems, such as nonlinear heat transfer, fluid mechanics, and many others, have been employed. Moatimid et al.^37^ employed an MHD flow of an incompressible nano liquid based on the non-Newtonian Jeffrey model in their study of motile microorganisms. By using the appropriate similarity transformations, the structural PDEs of motion, energy, nanoparticle volume fraction, and microbe intensity were converted into a set of nonlinear ODEs. Utilizing the HPM, analytically presented solutions were discovered. It was researched how an incompressible nanofluid moved after a non-Newtonian liquid. The Casson prototype defined the performance of the non-Newtonian liquid. The HPM was employed to systematically analyze the fundamental equations of motion.

The most important purpose of the current study is to investigate the symmetrically peristaltic movement of microorganisms that are common in RF in light of the aforementioned factors and due to the industrial significance of RF. The innovation of the present model derives from its important applications in the human body’s peristaltic channels through the digestive, urinary, and respiratory systems. The importance of recognizing and controlling the recognizing flow, nanoparticles, microbes, viruses, and bacteria with growing or reducing various parameters to increase the useful activities and decrease the harmful ones. The flow in a vertical tube with peristalsis and thermophoretic particle deposition is considered. Heat radiation and the properties of chemical reactions affect the temperature profile and volume concentration. The LWN and LRN approximations are applied to simplify the fundamental nonlinear PDEs. These equations are put through a series of non-dimensional transformations, producing a collection of ODEs that are not linear. The HPM is used to analyze the design of equational analytical solutions.

Therefore, this work is performed to yield answers to the following:

- How does the flow of microorganisms within an RF behave when flowing through a peristaltic path?
- What are the impacts of radiation, temperature sources, chemical reactions, and thermophoretic reactions deposition on the flow-related distributions?
- How are velocity, temperature, nanoparticles, and microorganism distributions work with the hypothetical conceptions?
- What is the basic significance of the persuaded parameters?

The rest of this manuscript is constructed as: Section “Modeling and solution of the structure” presents the approach of the problem, the applicable boundary conditions, the related physical coefficients, and the appropriate non-dimensional transforms. By utilizing the HPM, Section “Solution procedure” demonstrates the analytical solutions of the concluded boundary value problem. Section “Results and discussions” clarifies and discusses the results with some significant physical interpretations. The concluding observations and comments are presented in Section “Concluding remarks”.

## Modeling and solution of the structure

The current article aims to study the peristaltic transportation of a non-Newtonian two-dimensional RF containing fluctuating motile, gyrotactic microorganisms in the effect of heat radiation, heat source, chemical reaction, and thermophoretic particle deposition for nanoparticle movement. The flow is taken through a peristaltic sine-wavy channel. The Cartesian coordinate structure is taken into consideration, where the $X$- axis is united with the channel axis and the $Y$-axis is vertical. The geometry of channel walls^21^ is presented as:

1 $$Y = \pm h(X,t) = \pm \left( {a + b\,\sin \frac{2\pi }{\lambda }(X - c\,t)} \right)$$

**Figure 1:**
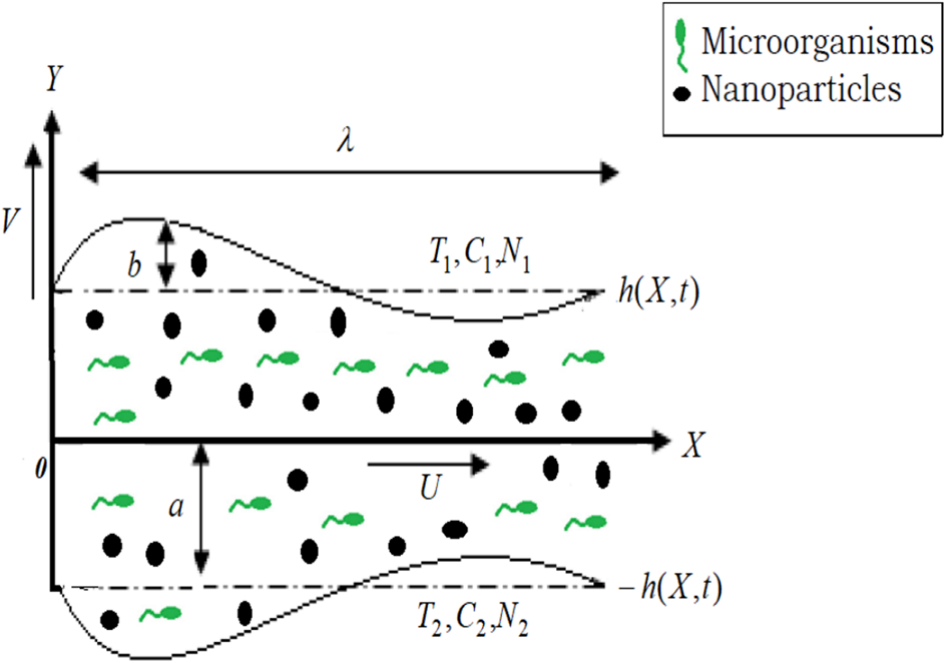
Depicts the theoretical model.

The channel limitations are maintained at uniform distinct temperatures, nanoparticles volume fraction, and microorganism concentrations. In accordance with the abovementioned construction, the prototype is explained and displayed in Fig. 1. The present model of flow has numerous significant and interesting medical, industrial, and engineering-relevant implications. There are a lot of vital processes related to the flow in peristaltic tubes, especially in humans and all living organism bodies like the journey of food through the digestive system. Additionally, many industrial machines and instruments depend on motion with peristalsis (peristaltic pumps) in their mechanism of work.

A peristaltic metering pump is a type of positive transportation pump, in which the fluid is pushed through a flexible pipe in a peristaltic motion form. Roller wheels are connected to a rotary part that is controlled by a motor. As the rotor revolves, the rollers squeeze the pipe to push the fluid forward. These kinds of pumps push unclean fluids that contain particulate matter like nanoparticles, microbes, and bacteria into lower-pressure systems. The smooth forces formed through the peristaltic pumping work do not damage the needed fluids within the pipe^38^ and^39^. Peristaltic pumps are used also in medical distillation pumps, dialysis (Kidney washing) machines, and open-heart pump machines. They are also useful for agriculture pumps, liquid food dispensers such as those for liquid cheese, pharmaceutical production, aggressive chemicals of all kinds and dosing systems, paint, pigments, printing, and washing machine fluid pumps.

### Basic movement formulas and relevant bounder restrictions

The shearing stress formula $\underline{\underline{S}}$ of the RF may be characterized by^20,21^ as follows:

2 $$\underline{\underline{S}} + \alpha^{*} \,\underline{\underline{S}}^{3} = \mu \,\underline{\underline{{\gamma^{.} }}}$$

The RF model behaves like the Newtonian fluid when $\alpha^{*} = 0$, and performs like pseudoplastic fluid when $\alpha^{*} > 0$, while it performs as a dilatant liquid when $\alpha^{*} < 0$.

In agreement with the above-stated standards, the fundamental formulae of continuity, as well as momentum equations with temperature, concentration and microorganism dispersions may be created as follows:

The continuity equation^21^ gives

3 $$\frac{\partial U}{{\partial X}} + \frac{\partial V}{{\partial Y}} = 0$$

The momentum formula in $X$-path^21,30,34^ gives

4 $$\left. \begin{gathered} \,\,\,\,\,\,\,\,\,\,\,\,\,\,\,\,\,\,\,\,\rho_{f} \left( {\frac{\partial U}{{\partial t}} + U\frac{\partial U}{{\partial X}} + {\text{V}}\frac{\partial U}{{\partial Y}}} \right) = - \frac{\partial p}{{\partial X}} + \frac{{\partial S_{XX} }}{\partial X} + \frac{{\partial S_{XY} }}{\partial Y} + \hfill \\ g\left[ {\rho_{f} \beta^{*} (1 - C_{2} )(T - T_{2} ) - (\rho_{p} - \rho_{f} )(C - C_{2} ) - \gamma (\rho_{m} - \rho_{f} )(N - N_{2} )} \right] \hfill \\ \end{gathered} \right\}.$$

The momentum equation in $Y$-direction^21^ yields

5 $$\rho_{f} \left( {\frac{{\partial {\text{V}}}}{\partial t} + U\frac{{\partial {\text{V}}}}{\partial X} + {\text{V}}\frac{{\partial {\text{V}}}}{\partial Y}} \right) = - \frac{\partial p}{{\partial Y}} + \frac{{\partial S_{YX} }}{\partial X} + \frac{{\partial S_{YY} }}{\partial Y}$$

The temperature formula in the existence of thermal radiation and temperature resource^40–42^ becomes

6 $$(\rho c)_{f} \left( {\frac{\partial T}{{\partial t}} + U\frac{\partial T}{{\partial X}} + {\text{V}}\frac{\partial T}{{\partial Y}}} \right) = k\left( {\frac{{\partial^{2} T}}{{\partial X^{2} }} + \frac{{\partial^{2} T}}{{\partial Y^{2} }}} \right) + \frac{{16\sigma^{*} T_{2}^{3} }}{{3k^{*} }}\left[ {\frac{{\partial^{2} T}}{{\partial X^{2} }} + \frac{{\partial^{2} T}}{{\partial Y^{2} }}} \right] + Q_{1} \left( {T - T_{2} } \right)$$

The nanoparticle concentration formula with the impact of thermophoretic velocity and chemical reaction^5,35^ and^40^ gives

7 $$\frac{\partial C}{{\partial t}} + U\frac{\partial C}{{\partial X}} + {\text{V}}\frac{\partial C}{{\partial Y}} = \,D_{B} \left( {\frac{{\partial^{2} C}}{{\partial X^{2} }} + \frac{{\partial^{2} C}}{{\partial Y^{2} }}} \right) - \nabla .\left[ {V_{T} (C - C_{2} )} \right] - R_{1} (C - C_{2} )$$

where $$V_{T} = - \frac{{k^{*} \upsilon }}{{T_{2} }}\nabla T$$.

Additionally, the microorganism diffusion equation^31^ and^35^ provides

8 $$\frac{\partial N}{{\partial t}} + U\frac{\partial N}{{\partial X}} + {\text{V}}\frac{\partial N}{{\partial Y}} + \frac{{\overline{b}\,W_{c} }}{\Delta C}\left( {\frac{\partial }{\partial X}\left( {N\frac{\partial C}{{\partial X}}} \right) + \frac{\partial }{\partial Y}\left( {N\frac{\partial C}{{\partial Y}}} \right)} \right) = \,D_{m} \left( {\frac{{\partial^{2} N}}{{\partial X^{2} }} + \frac{{\partial^{2} N}}{{\partial Y^{2} }}} \right).$$

The investigation at hand is restricted by the preceding formulae. They must get together a number of border requirements. The necessary boundary criteria^22^ can be identified as follows:

9 $$\left. {\begin{array}{*{20}l} {U = 0,\,T = T_{1} ,\,C = C_{1} \,{\text{and}}\,N = N_{1} } \hfill & {{\text{at}}\,\,\,\,Y = h(X,t)} \hfill \\ {\frac{\partial U}{{\partial Y}} = 0,} \hfill & {{\text{at}}\,\,\,\,\,\,{\text{Y}} = {0}} \hfill \\ {T = T_{2} ,\,C = C_{2} \,{\text{and}}\,N = N_{2} } \hfill & {{\text{at}}\,\,\,\,\,Y = - h(X,t)} \hfill \\ \end{array} } \right\}.$$

To begin a wave construction $(x,y)$ which changes with speed $c$ outside the given border, the following transformations are suggested as^20,24^, and^40^:

10 $$x = X - c\,t,\,\,\,y = Y,\,\,\,u = U - c\,\,\,{\text{and}}\;\;\;{\text{v}} = {\text{V}}$$

Accordingly, Eqs. (3)–(8) become:

11 $$ \frac{\partial \rm u}{{\partial \rm x}} + \frac{\partial \rm v}{{\partial \rm y}} = 0 $$

12 $$\left. \begin{gathered} \,\,\,\,\,\,\,\,\,\,\,\,\,\,\,\,\,\,\,\,\,\,\,\,\,\,\,\,\,\,\,\,\,\,\,\rho_{f} \left( {u\frac{\partial u}{{\partial x}} + {\text{v}}\frac{\partial u}{{\partial y}}} \right) = - \frac{\partial p}{{\partial x}} + \frac{{\partial S_{xx} }}{\partial x} + \frac{{\partial S_{xy} }}{\partial y} + \hfill \\ g\left[ {\rho_{f} \beta^{*} (1 - C_{2} )(T - T_{2} ) - (\rho_{p} - \rho_{f} )(C - C_{2} ) - \gamma (\rho_{m} - \rho_{f} )(N - N_{2} )} \right] \hfill \\ \end{gathered} \right\}$$

13 $$\rho_{f} \left( {u\frac{{\partial {\text{v}}}}{\partial x} + {\text{v}}\frac{{\partial {\text{v}}}}{\partial y}} \right) = - \frac{\partial p}{{\partial \overline{y}}} + \frac{{\partial \tau_{yx} }}{\partial x} + \frac{{\partial \tau_{yy} }}{\partial y}$$

14 $$(\rho C)_{f} \left( {u\frac{\partial T}{{\partial x}} + {\text{v}}\frac{\partial T}{{\partial y}}} \right) = k\left( {\frac{{\partial^{2} T}}{{\partial x^{2} }} + \frac{{\partial^{2} T}}{{\partial y^{2} }}} \right) + \frac{{16\sigma^{*} T_{2}^{3} }}{{3k^{*} }}\left[ {\frac{{\partial^{2} T}}{{\partial x^{2} }} + \frac{{\partial^{2} T}}{{\partial y^{2} }}} \right] + Q_{1} \left( {T - T_{2} } \right)$$

15 $$u\frac{\partial C}{{\partial x}} + {\text{v}}\frac{\partial C}{{\partial y}} = \,D_{B} \left( {\frac{{\partial^{2} C}}{{\partial x^{2} }} + \frac{{\partial^{2} C}}{{\partial y^{2} }}} \right) + \frac{{k^{*} \upsilon }}{{T_{2} }}\left[ {\left( {\frac{\partial T}{{\partial x}}\frac{\partial C}{{\partial x}} + \frac{\partial T}{{\partial y}}\frac{\partial C}{{\partial y}}} \right) + (C - C_{2} )\left( {\frac{{\partial^{2} T}}{{\partial x^{2} }} + \frac{{\partial^{2} T}}{{\partial y^{2} }}} \right)} \right] - R_{1} (C - C_{2} )$$

Additionally,

16 $$u\frac{\partial N}{{\partial x}} + {\text{v}}\frac{\partial N}{{\partial y}} + \frac{{\overline{b}\,W_{c} }}{\Delta C}\left( {\frac{\partial }{\partial x}\left( {N\frac{\partial C}{{\partial x}}} \right) + \frac{\partial }{\partial y}\left( {N\frac{\partial C}{{\partial y}}} \right)} \right) = \,D_{m} \left( {\frac{{\partial^{2} N}}{{\partial x^{2} }} + \frac{{\partial^{2} N}}{{\partial y^{2} }}} \right)$$

with the border criteria

17 $$\left. \begin{gathered} u = - c,\,T = T_{1} ,\,C = C_{1} \,{\text{and}}\,N = N_{1} \,\,\,\,\,\,{\text{at}}\,\,\,\,\,y = h(x) \hfill \\ \frac{\partial u}{{\partial y}} = 0,\,\,\,\,\,\,\,{\text{at}}\,\,\,\,\,y = 0 \hfill \\ T = T_{2} ,\,C = C_{2} \,{\text{and}}\,N = N_{2} \,\,\,\,\,\,{\text{at}}\,\,\,\,\,y = - h(x) \hfill \\ \end{gathered} \right\}$$

Finally, the problem at this point has been well established. The solution will be crystallized in Section “Results and discussions”.

### Significant physiological concepts

The tremendous amount of attention in this analysis is concerned with the skin friction factor, which is the outcome of the viscosity of the fluid through which it passes, and is defined as:

18 $$C_{f} = \left. {S_{xy} } \right|_{y = h} /\rho_{f} v^{2}$$

whereas the Nusselt numeral, the Sherwood numeral, and the Motile numeral are numbers required only within the framework of the boundary layer theory. So, this investigation emphasizes the discussion of the skin friction coefficient.

### Appropriate dimensionless amounts

The dimensionless method may be used to reduce quantities by using measurable units. The appropriate dimensionless quantities^21^ and^34^ may be written as follows:

19 $$\left. \begin{gathered} x^{*} = \frac{x}{\lambda },\,y^{*} = y/a,\,u^{*} = u/c,\,{\text{v}}^{*} = {\text{v}}/c,\,h^{*} = h/a,\,p^{*} = a\,p/\mu \,c,Re = \frac{{\rho_{f} c\,a}}{\mu } \hfill \\ \,\,\,\delta = \frac{a}{\lambda },\,S_{ij}^{*} = S_{ij} a/\mu \,c,\theta = \frac{{T - T_{2} }}{{T_{1} - T_{2} }},\,\phi = \frac{{C - C_{2} }}{{C_{1} - C_{2} }},\,\,\,\,{\text{and}}\,\,\chi = \frac{{N - N_{2} }}{{N_{1} - N_{2} }} \hfill \\ \end{gathered} \right\}$$

The non-dimensional variables (19) are inserted into both of the constitutive equation of RF (2) and the main formulae of motion as shown in Eqs. (11)–(16), with the border restrictions (17) and the skin friction parameter (18). The star mark is deleted for simplicity, and both the LWL $(\delta < < 1)$ and SRN approximations^17^ are expected. Therefore, the main formulae scheming the fluid flow can be reformulated as follows:

20 $$\frac{\partial P}{{\partial x}} = \frac{{\partial S_{xy} }}{\partial y} + Gr\left( {\theta - N_{r} \,\phi - R_{b} \,\chi } \right)$$

21 $$\frac{\partial P}{{\partial y}} = 0$$

22 $$S_{xy} + \alpha \,S_{xy}^{3} = \frac{\partial u}{{\partial y}}$$

23 $$\,\frac{{\partial^{2} \theta }}{{\partial y^{2} }} + R_{d} \frac{{\partial^{2} \theta }}{{\partial y^{2} }} + Q\Pr \theta = 0$$

24 $$\,\frac{{\partial^{2} \phi }}{{\partial y^{2} }} - \Gamma Sc\,\left( {\frac{{\partial^{2} \theta }}{{\partial y^{2} }}\phi + \frac{\partial \phi }{{\partial y}}\frac{\partial \theta }{{\partial y}}} \right) - R\,Sc\,\phi = 0$$

25 $${\text{and}}\;\;\frac{{\partial^{2} \chi }}{{\partial y^{2} }} = Pe\left( {\frac{\partial \chi }{{\partial y}}\frac{\partial \phi }{{\partial y}} + (\chi + \sigma )\frac{{\partial^{2} \phi }}{{\partial y^{2} }}} \right)$$

Additionally, the boundary circumstances become:

26 $$\left. \begin{gathered} u = - 1,\,\theta = \,\phi = \chi = 1\,\,\,\,\,\,{\text{at}}\,\,\,\,\,y = h(x) \hfill \\ \frac{\partial u}{{\partial y}} = 0,\,\,\,\,\,\,\,{\text{at}}\,\,\,\,\,y = 0 \hfill \\ \theta = \,\phi = \chi = 0\,\,\,\,\,\,{\text{at}}\,\,\,\,\,y = - h(x) \hfill \\ \end{gathered} \right\}$$

Also, the skin friction parameter becomes:

27 $$C_{f} = \left. {S_{xy} } \right|_{y = h}$$

where for more convenience, all physical factors in the dimensionless structure can be communicated as follows:

$$Gr = g\beta^{*} (1 - C_{2} )(T_{1} - T_{2} )a^{2} /c\mu$$, $$N_{r} = g(\rho_{p} - \rho_{f} )(C_{1} - C_{2} )a^{2} /\rho_{f} c\mu$$, $$\alpha = \frac{{\mu^{2} c^{2} }}{{a^{2} }}\alpha^{*}$$, $$R_{b} = g\gamma (\rho_{m} - \rho_{f} )(N_{1} - N_{2} )a^{2} /\rho_{f} c\mu$$, $$R_{d} = \frac{{16\sigma^{*} T_{2}^{3} }}{{3kk^{*} }}$$, $$\Pr = \upsilon (\rho C)_{f} /k$$, $$\Gamma = - k^{*} (T_{1} - T_{2} )/T_{2}$$, $$Sc = \upsilon /D_{B}$$, $$R = R_{1} a^{2} /\upsilon$$, $$\sigma = N_{2} /(N_{1} - N_{2} )$$ and $$Pe = b\,Wc/D_{m}$$.

At this stage, it should be noted that the problem formulation as indicated by Eqs. (20)–(25) will be the production of^20^ for $Gr = R = \chi = R_{d} = 0$.

### Solution procedure

The analytical solution of the boundary value problem (23)–(25), utilizing the appropriate boarder criteria (26), was obtained by using the HPM^36^ and^37^. He^36^ officially is the first to solve an ODE by establishing an artificial incorporated factor $q \in \left[ {0,\,1} \right]$ into the PDEs. The HPM is among the noteworthy novel methods for resolving both linear and nonlinear PDES. The following formulation may be employed to solve the abovementioned formulae:

28 $$H(\theta ,q) = \,\frac{{\partial^{2} \theta }}{{\partial y^{2} }} + q\left[ {\frac{Q\Pr }{{\left( {1 + R_{d} } \right)}}\theta } \right] = 0$$

29 $$H(\phi ,q) = \,\frac{{\partial^{2} \phi }}{{\partial y^{2} }} + q\left[ { - \Gamma Sc\,\left( {\frac{{\partial^{2} \theta }}{{\partial y^{2} }}\phi + \frac{\partial \phi }{{\partial y}}\frac{\partial \theta }{{\partial y}}} \right) - R\,Sc\,\phi } \right] = 0$$

30 $$H(\chi ,q) = \frac{{\partial^{2} \chi }}{{\partial y^{2} }} + q\left[ { - Pe\left( {\frac{\partial \chi }{{\partial y}}\frac{\partial \phi }{{\partial y}} + (\chi + \sigma )\frac{{\partial^{2} \phi }}{{\partial y^{2} }}} \right)} \right] = 0$$

In the view of the preceding procedure, the dependent functions $\theta ,\,\phi$ and $\chi$ are inserted in the following equation instead of $\beta (y,q)$:

31 $$\beta (y,q) = \beta_{0} (y) + q\beta_{1} (y) + q^{2} \beta_{2} (y) + ...$$

Inserting Eq. (31) into Eqs. (28)–(30) and the appropriate border criteria (26) with equating the similar exponents of $q$- terms, we get the zero order equations as follows:

32 $$\frac{{\partial^{2} \psi }}{{\partial y^{2} }} = 0$$

where $\psi$ stands for the functions $\theta_{0} ,\,\phi_{0}$ and $\chi_{0}$, with the border restrictions:

33 $$\left. \begin{gathered} \theta_{0} = \,\phi_{0} = \chi_{0} = 1\,\,\,\,\,\,{\text{at}}\,\,\,\,\,\,y = h(x) \hfill \\ \,\theta_{0} = \,\phi_{0} = \chi_{0} = 0\,\,\,\,\,\,{\text{at}}\,\,\,\,\,y = - h(x) \hfill \\ \end{gathered} \right\}$$

The first order equations are as follows:

34 $$\frac{{\partial^{2} \theta_{1} }}{{\partial y^{2} }} + \frac{\Pr Q}{{1 + R_{d} }}\theta_{0} = 0$$

35 $$\frac{{\partial^{2} \phi_{1} }}{{\partial y^{2} }} - \Gamma Sc\,\left( {\frac{{\partial^{2} \theta_{0} }}{{\partial y^{2} }}\phi_{0} + \frac{{\partial \phi_{0} }}{\partial y}\frac{{\partial \theta_{0} }}{\partial y}} \right) - R_{c} \,Sc\,\phi_{0} = 0$$

36 $$\frac{{\partial^{2} \chi_{1} }}{{\partial y^{2} }} - Pe\left( {\frac{{\partial \chi_{0} }}{\partial y}\frac{{\partial \phi_{0} }}{\partial y} + (\chi_{0} + \sigma )\frac{{\partial^{2} \phi_{0} }}{{\partial y^{2} }}} \right) = 0$$

with the boundary conditions:

37 $$\left. \begin{gathered} \,\theta_{0} = \,\phi_{0} = \chi_{0} = 0\,\,\,\,\,\,{\text{at}}\,\,\,\,\,y = h(x) \hfill \\ \theta_{0} = \,\phi_{0} = \chi_{0} = 0\,\,\,\,\,\,{\text{at}}\,\,\,\,\,y = - h(x) \hfill \\ \end{gathered} \right\}$$

and the second order equations are as follows:

38 $$\frac{{\partial^{2} \theta_{2} }}{{\partial y^{2} }} + \frac{\Pr Q}{{1 + R_{d} }}\theta_{1} = 0$$

39 $$\frac{{\partial^{2} \phi_{2} }}{{\partial y^{2} }} - \Gamma Sc\,\left( {\phi_{1} \frac{{\partial^{2} \theta_{0} }}{{\partial y^{2} }} + \phi_{0} \frac{{\partial^{2} \theta_{1} }}{{\partial y^{2} }} + \frac{{\partial \phi_{0} }}{\partial y}\frac{{\partial \theta_{1} }}{\partial y} + \frac{{\partial \phi_{1} }}{\partial y}\frac{{\partial \theta_{0} }}{\partial y}} \right) - R_{c} \,Sc\,\phi_{1} = 0$$

40 $$\frac{{\partial^{2} \chi_{2} }}{{\partial y^{2} }} - Pe\left( {\frac{{\partial \chi_{1} }}{\partial y}\frac{{\partial \phi_{0} }}{\partial y} + \frac{{\partial \chi_{0} }}{\partial y}\frac{{\partial \phi_{1} }}{\partial y} + \chi_{1} \frac{{\partial^{2} \phi_{0} }}{{\partial y^{2} }} + (\chi_{0} + \sigma )\frac{{\partial^{2} \phi_{1} }}{{\partial y^{2} }}} \right) = 0$$

with the boundary conditions:

41 $$\left. \begin{gathered} \,\theta_{2} = \,\phi_{2} = \chi_{2} = 0\,\,\,\,\,\,{\text{at}}\,\,\,\,\,y = h(x) \hfill \\ \theta_{2} = \,\phi_{2} = \chi_{2} = 0\,\,\,\,\,\,{\text{at}}\,\,\,\,\,y = - h(x) \hfill \\ \end{gathered} \right\}$$

Consequently, the profiles of the functions $\theta \,,\,\phi$ and $\chi$, when $q \to 1$ in Eq. (31), can be represented as follows:

42 $$\theta (x,y) = a_{1} + a_{2} y + a_{3} y^{2} + a_{4} y^{3} + a_{5} y^{4} + a_{6} y^{5}$$

43 $$\phi (x,y) = a_{7} + a_{8} y + a_{9} y^{2} + a_{10} y^{3} + a_{11} y^{4} + a_{12} y^{5}$$

44 $$\chi (x,y) = a_{13} + a_{14} y + a_{15} y^{2} + a_{16} y^{3} + a_{17} y^{4}$$

For an easy follow up of the paper, the arithmetic expressions of $a_{1} - a_{17}$ are not included at this point. However, they are available in the Appendix Section, which is can be found as supplementary information link at the end of the article.

Substituting Eqs. (42)–(44) into Eq. (20), the shearing stress $S_{xy}$ along with the necessary boundary form:

45 $$S_{xy} = 0\;\;at\;\;\;y = 0$$

which can be directly formulated as follows:

46 $$S_{xy} = b_{1} y + b_{2} y^{2} + b_{3} y^{3} + b_{4} y^{4} + b_{5} y^{5} + b_{6} y^{6}$$

where $b_{1} ,\,b_{2} ,.......,b_{6}$ are listed in the Appendix Section, which is can be found as supplementary information link at the end of the article.

In conclusion, the expression of velocity profile from the constitutive Eq. (22) with regard to the border criteria is specified in Eq. (26) as:

47 $$u(x,y) = a_{18} + a_{19} y^{2} + a_{20} y^{3} + a_{21} y^{4} + \cdots \cdots + a_{36} y^{19}$$

The constants $a_{18} - a_{36}$ are also included in the Appendix Section, which is can be found as supplementary information link at the end of the article. Finally, impacts of the different parameters, that control this investigation, on velocity, temperature, nanoparticles, and microorganism profiles will be shown in next section with a number of tables and diagrams for more in-depth explanation.

## Results and discussions

The movement of a nano liquid obeying the non- Newtonian RF archetype across a peristaltic horizontal pipe under a constant pressure gradient is examined in the present work. Heat transfer and nano-particles volume fraction as well as the microorganism concentration distributions are considered, together with the effect of heat radiation, heat source, chemical response properties, and thermophoretic particle deposition. The non-dimensional differential Eqs. (20)–(25) with the border circumstances (26) are solved with the support of the HPM. The existing flow is considerable in numerous bio and medical purposes such as the liquids and food transmission inside the human body, which are considered as a symmetrical peristaltic movement in its healthful state. It is also applicable to the structure of some therapeutic surgical instruments like endoscopes. Moreover, the peristaltic motion is applicable in several manufacturing and production tools like peristaltic pumps as previously revealed.

**Figure 2:**
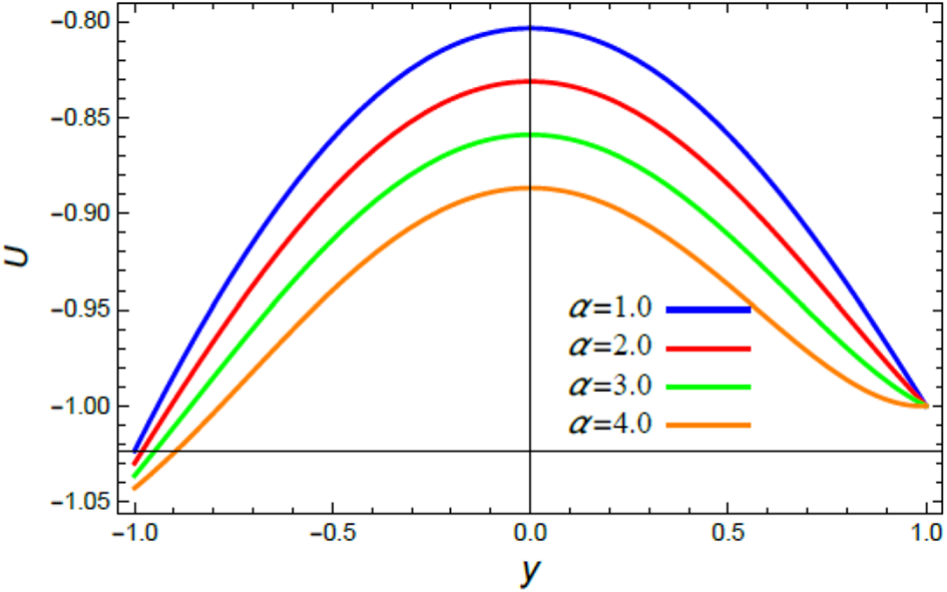
The effect of the pseudo plastic coefficient $\alpha$ on the speed distribution $u$.

**Figure 3:**
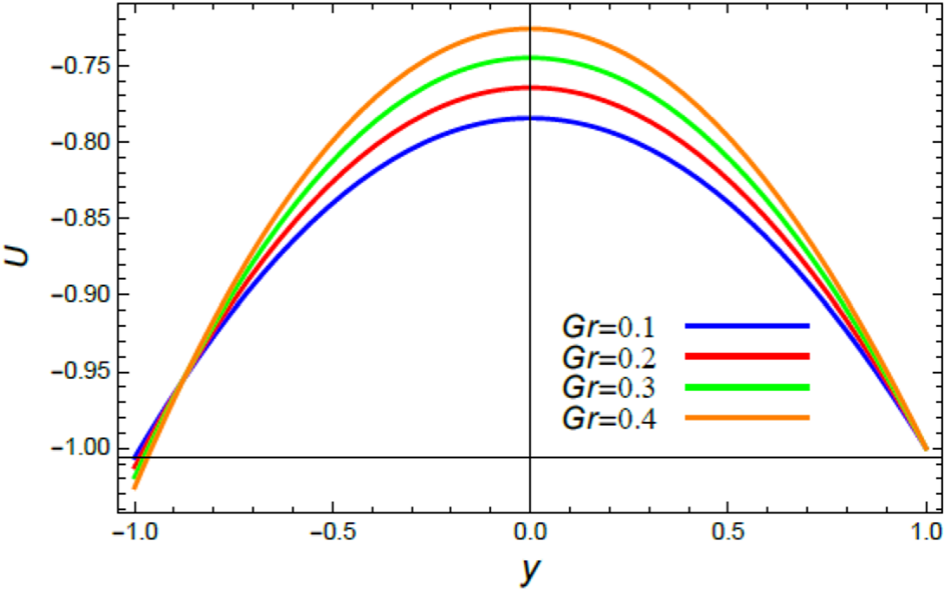
The influence of the Grashof numeral $Gr$ on the speed profile $u$.

**Figure 4:**
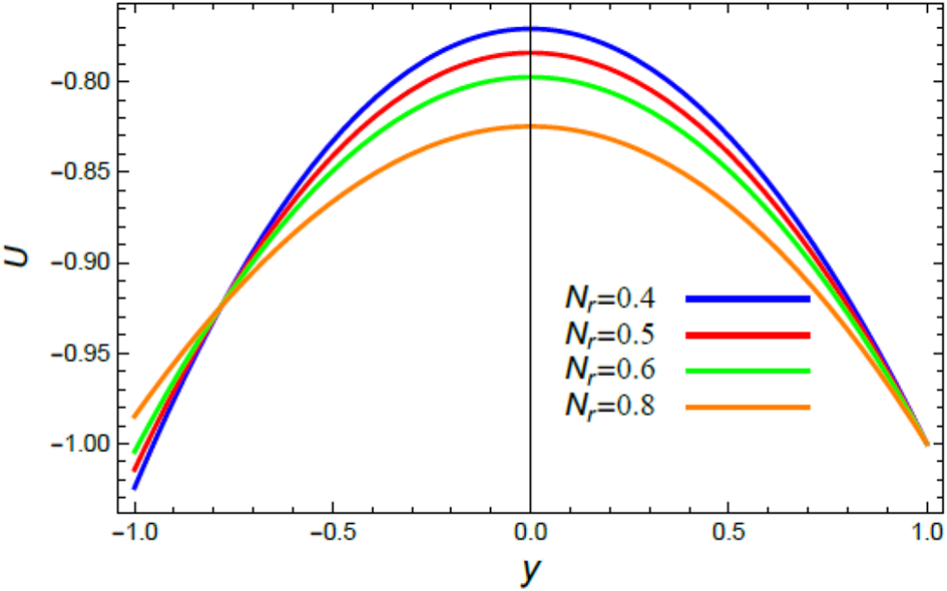
The impact of the buoyancy ratio factor $N_{r}$ on the speed distribution $u$.

**Figure 5:**
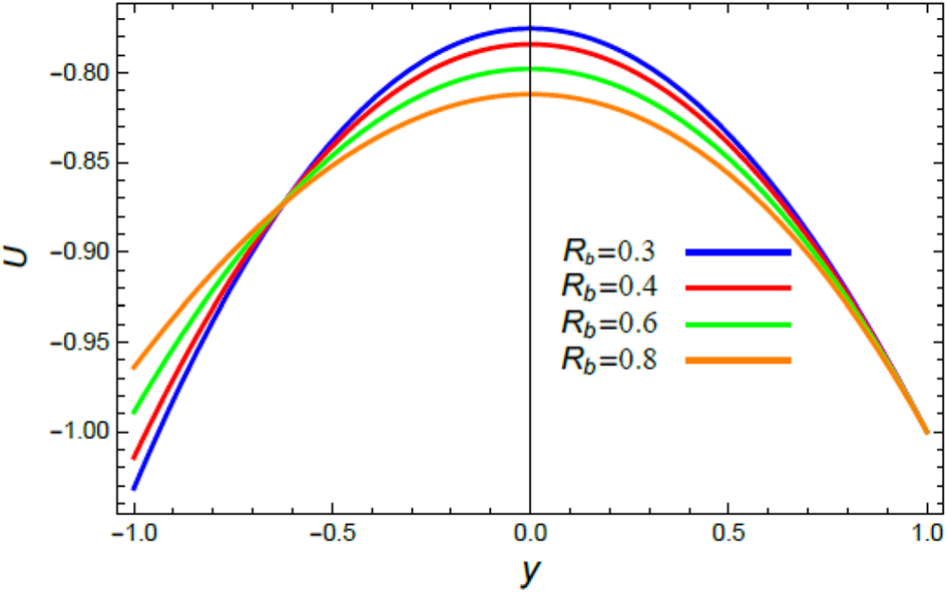
The impact of the bioconvection Rayleigh numeral $R_{b}$ on the speed profile $u$.

**Figure 6:**
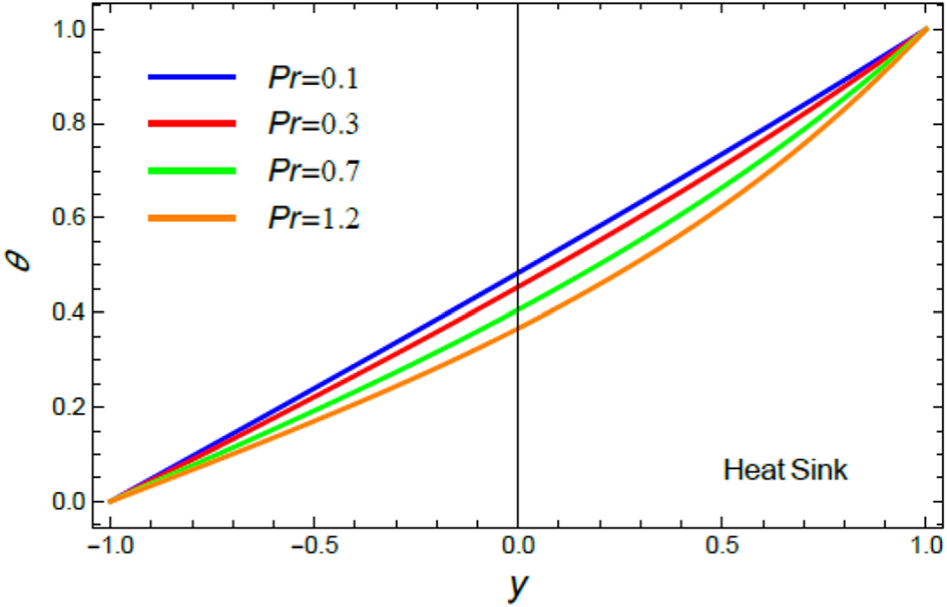
The impact of the Prandtl numeral $\Pr$ in the case of heat sink on the temperature profile $\theta$.

**Figure 7:**
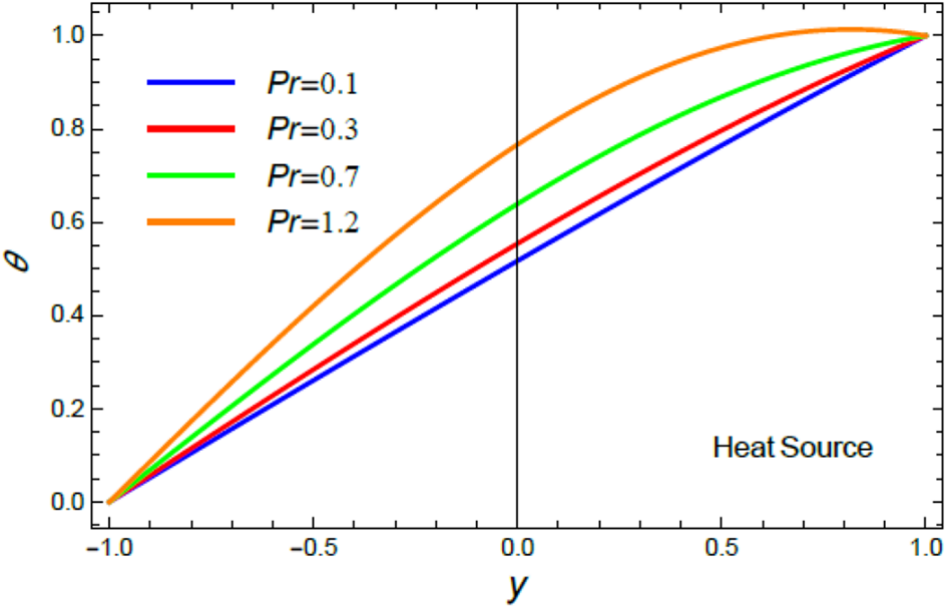
The impact of the Prandtl numeral $\Pr$ in the case of heat source on the heat profile $\theta$.

**Figure 8:**
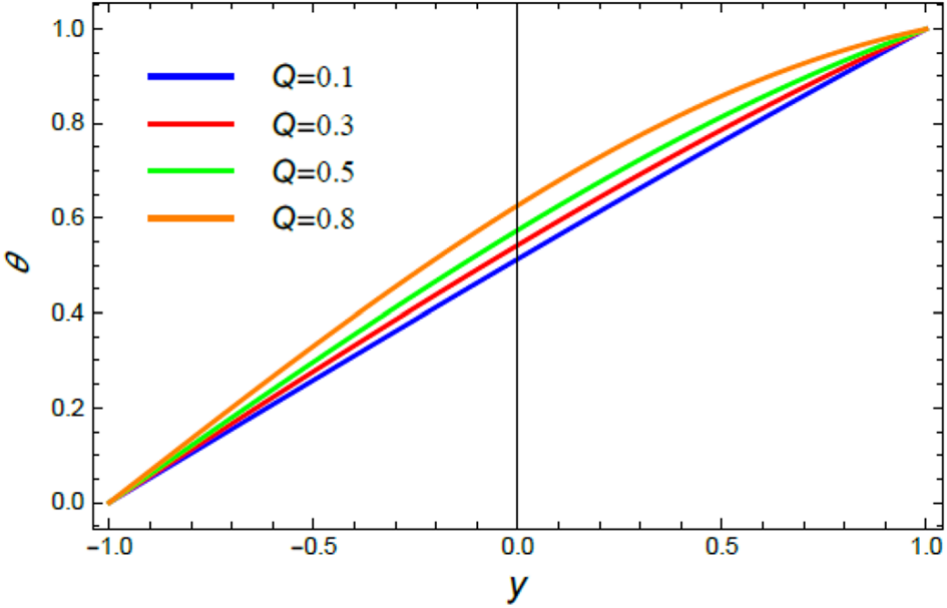
The effect of the heat resource coefficient $Q$ on the heat distribution $\theta$.

**Figure 9:**
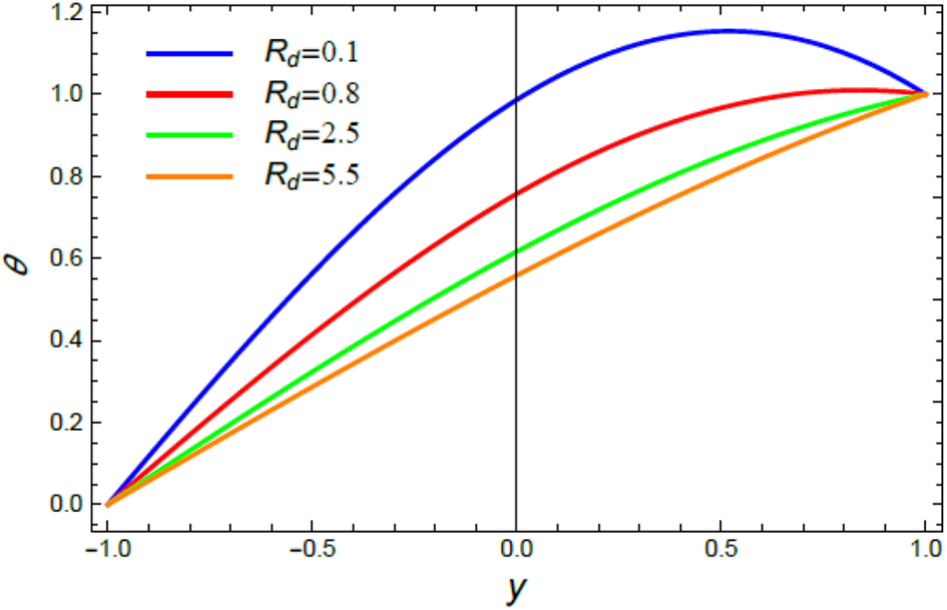
The impact of the radiation coefficient $R_{d}$ on the heat distribution $\theta$.

**Figure 10:**
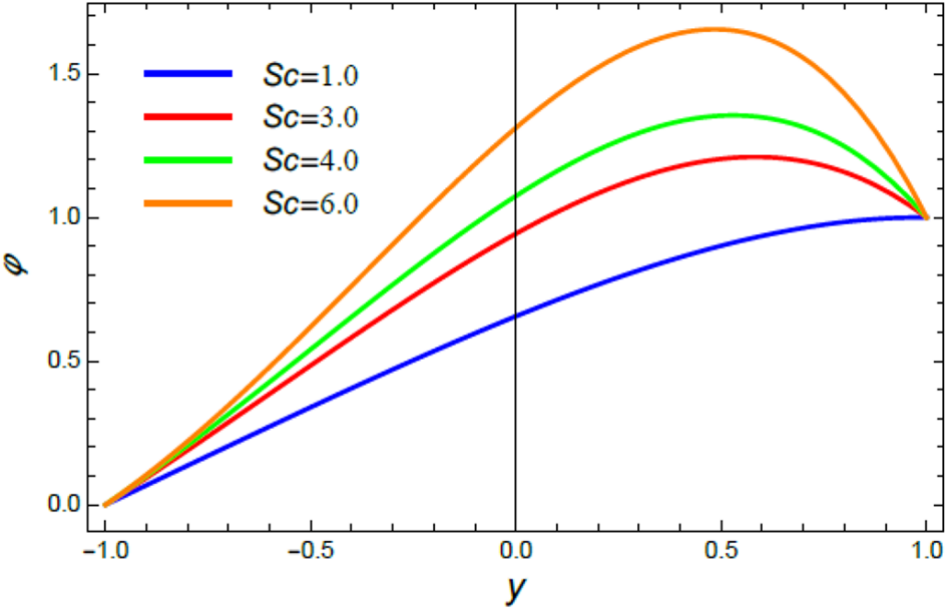
The nano-particles profile $\phi$ for various amounts of the Schmidt numeral $Sc$.

**Figure 11:**
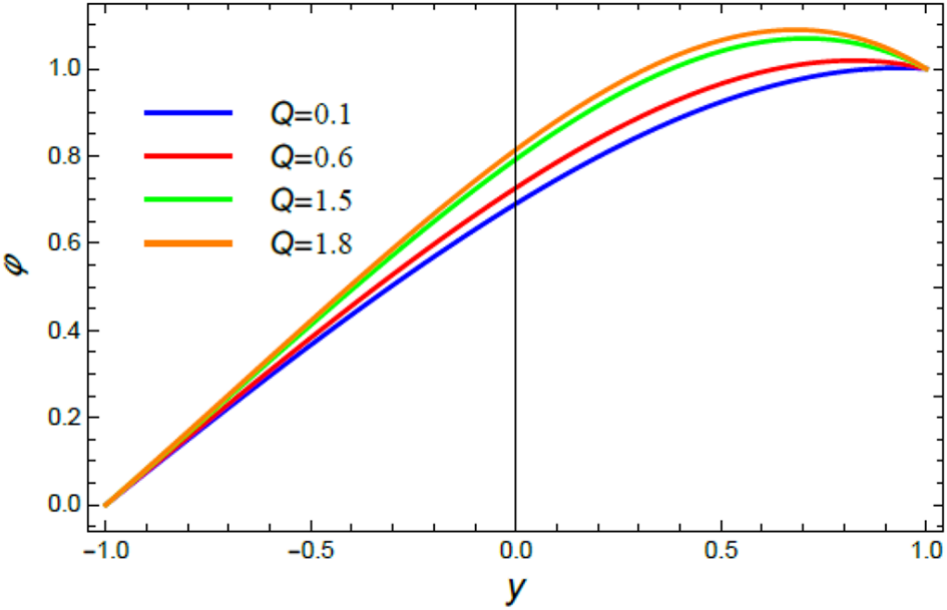
The nano-particles profile $\phi$ for various amounts of the heat source coefficient $Q$.

**Figure 12:**
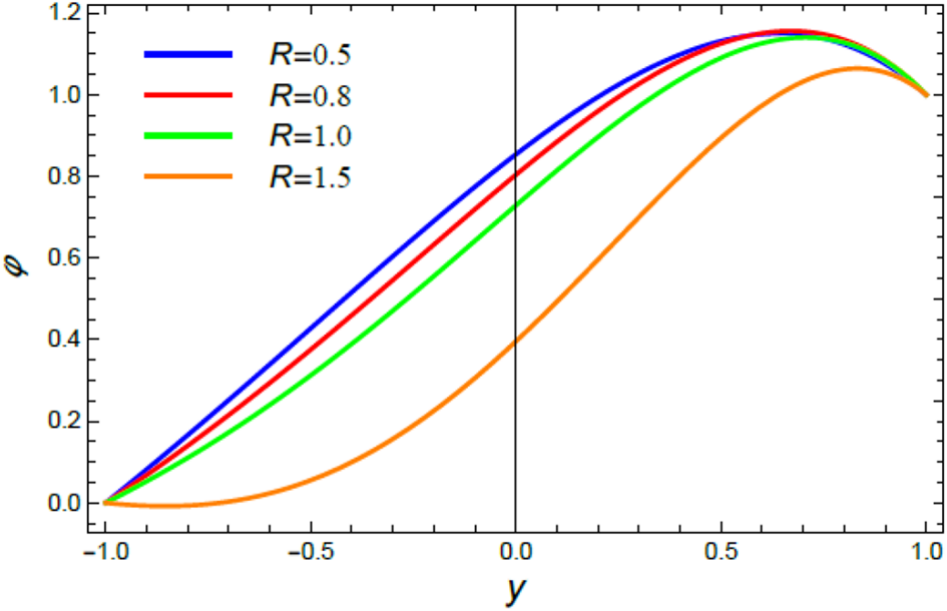
The nano-particles distribution $\phi$ for various amounts of the chemical reaction coefficient $R$.

**Figure 13:**
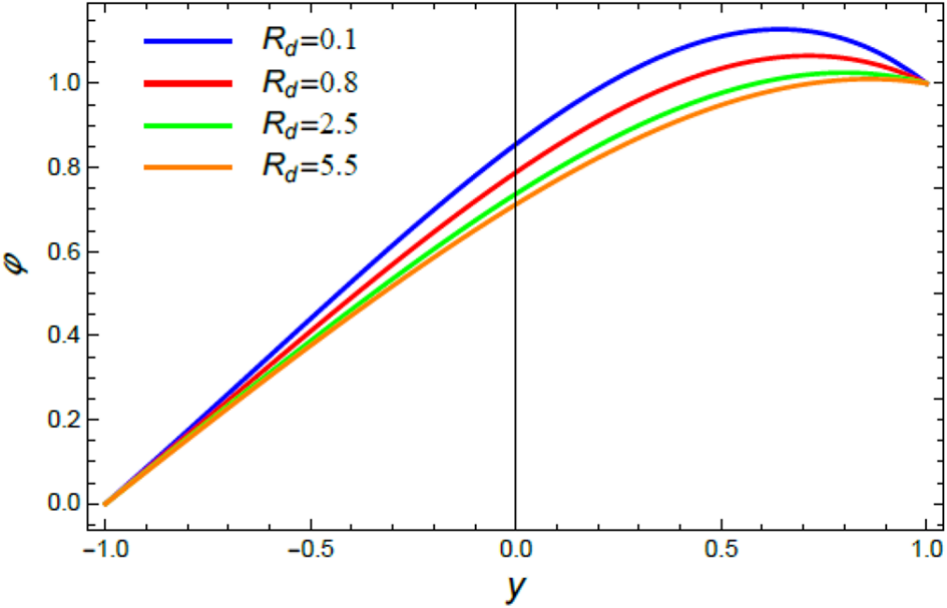
The nano-particles distribution $\phi$ for various amounts of the radiation parameter $R_{d}$.

**Figure 14:**
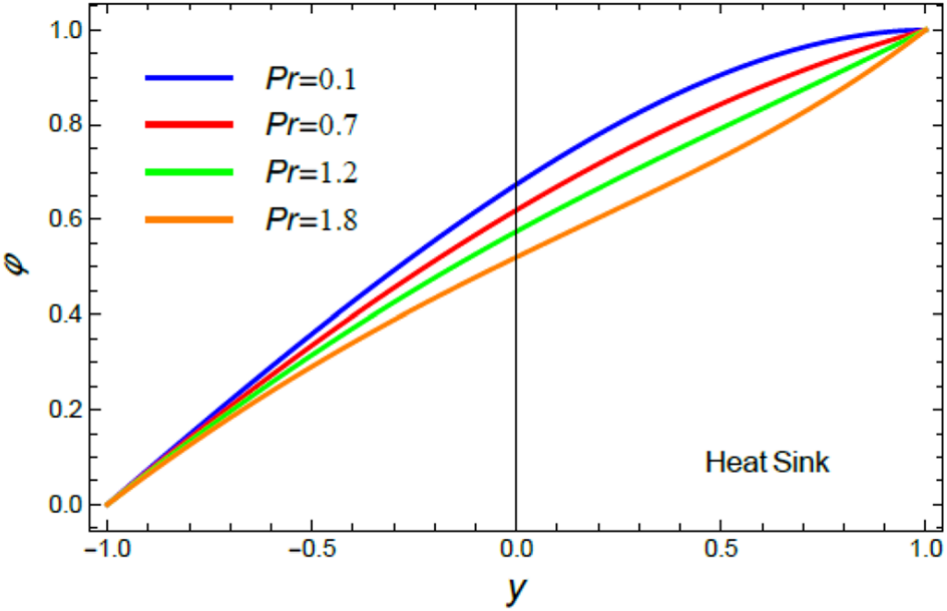
The nano-particles profile $\phi$ in the case of heat sink for different amounts of the Prandtl numeral $\Pr$.

**Figure 15:**
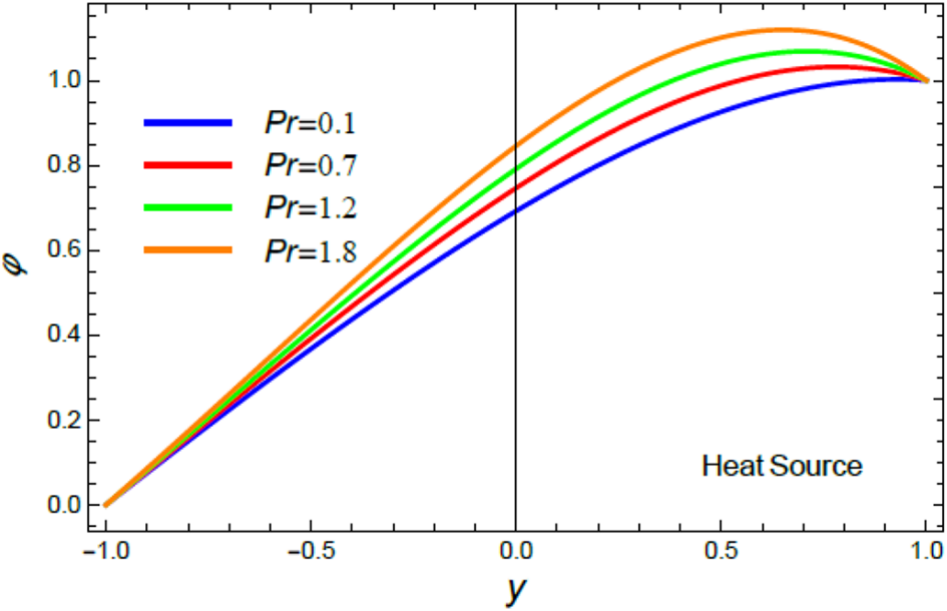
The nano-particles profile $\phi$ in the case of heat source for different amounts of the Prandtl numeral $\Pr$.

**Figure 16:**
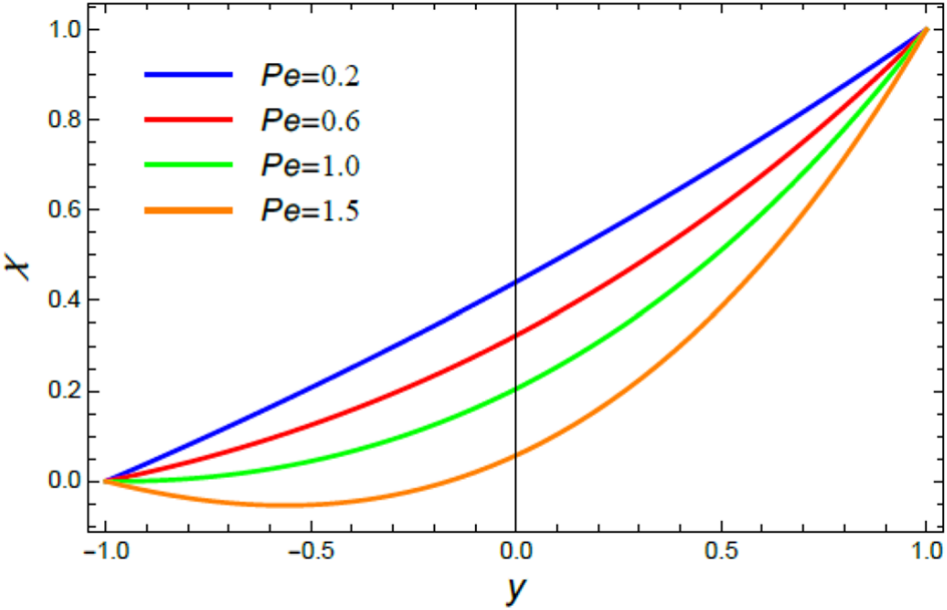
The microorganisms distribution $\chi$ for different amounts of the Peclet numeral $Pe$.

**Figure 17:**
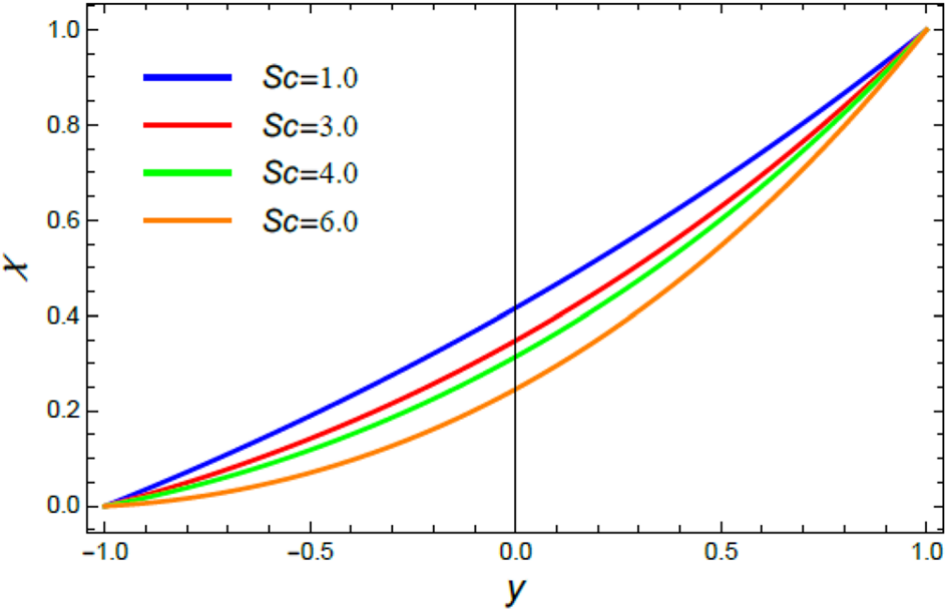
The Microorganisms profile $\chi$ for various amounts of the Schmidt numeral $Sc$.

**Figure 18:**
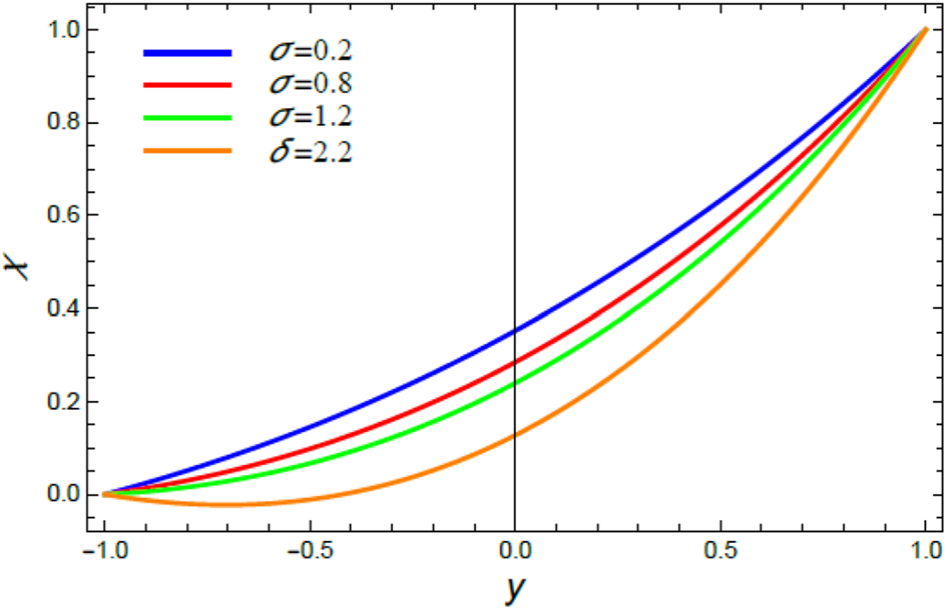
The microorganisms distribution $\chi$ for different amounts of the bioconvection constant $\sigma$.

**Figure 19:**
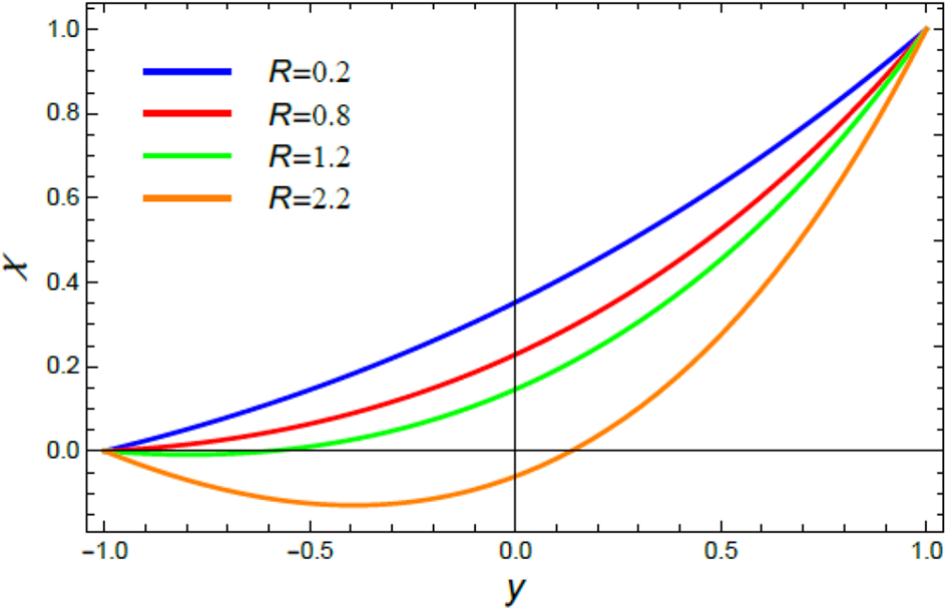
The microorganisms distribution $\chi$ for various amounts of the chemical reaction coefficient $R$.

**Figure 20:**
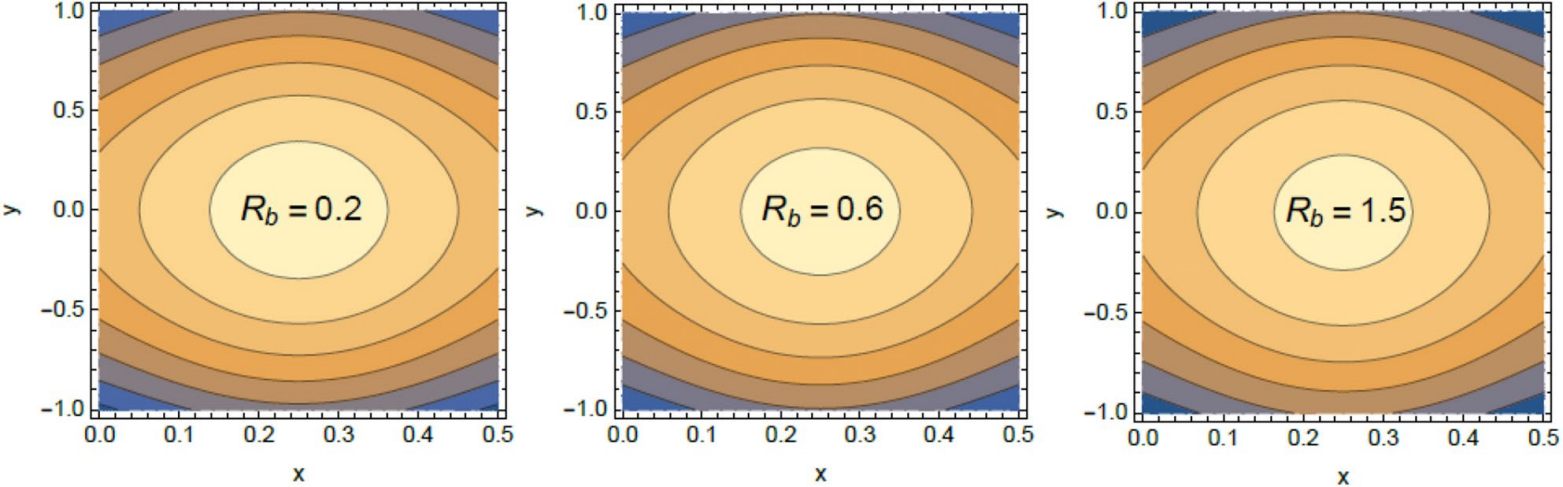
Contour plot of the velocity for several amounts of the bioconvection Rayleigh numeral $R_{b}$.

**Figure 21:**
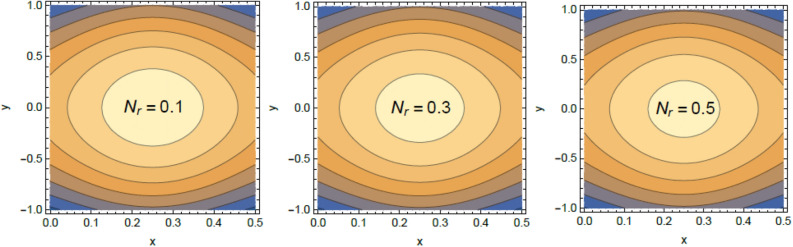
Contour plot of the speed for several amounts of the buoyancy ratio numeral $N_{r}$.

**Figure 22:**
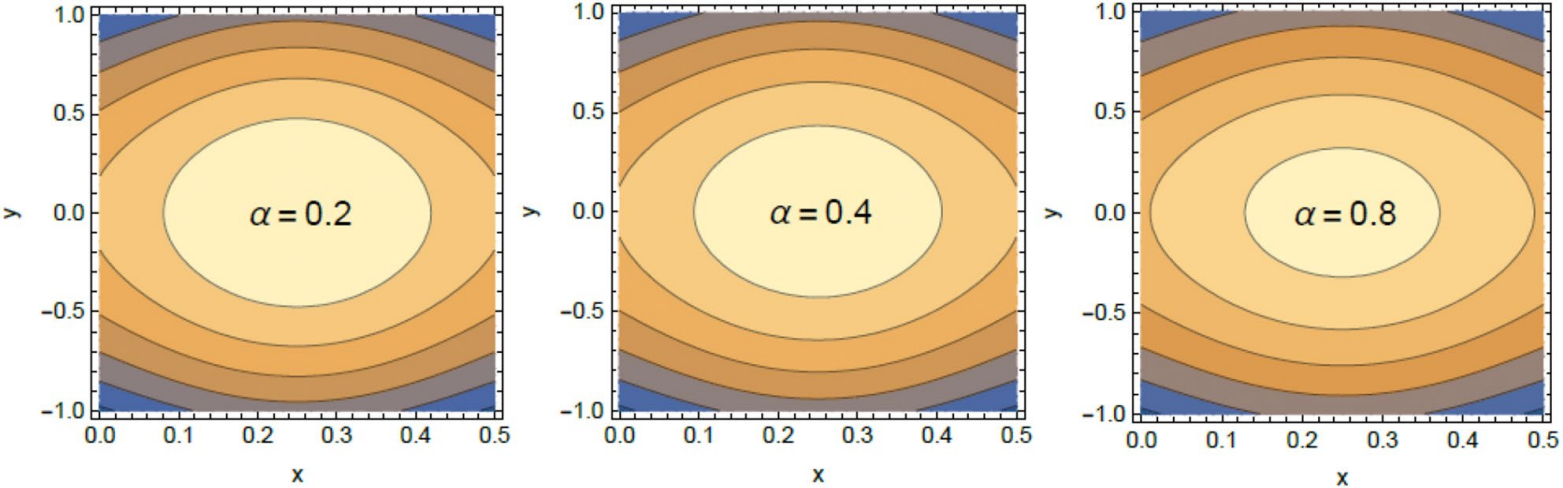
Contour plot of the speed for various amounts of the pseudo plastic factor $\alpha$.

**Figure 23:**
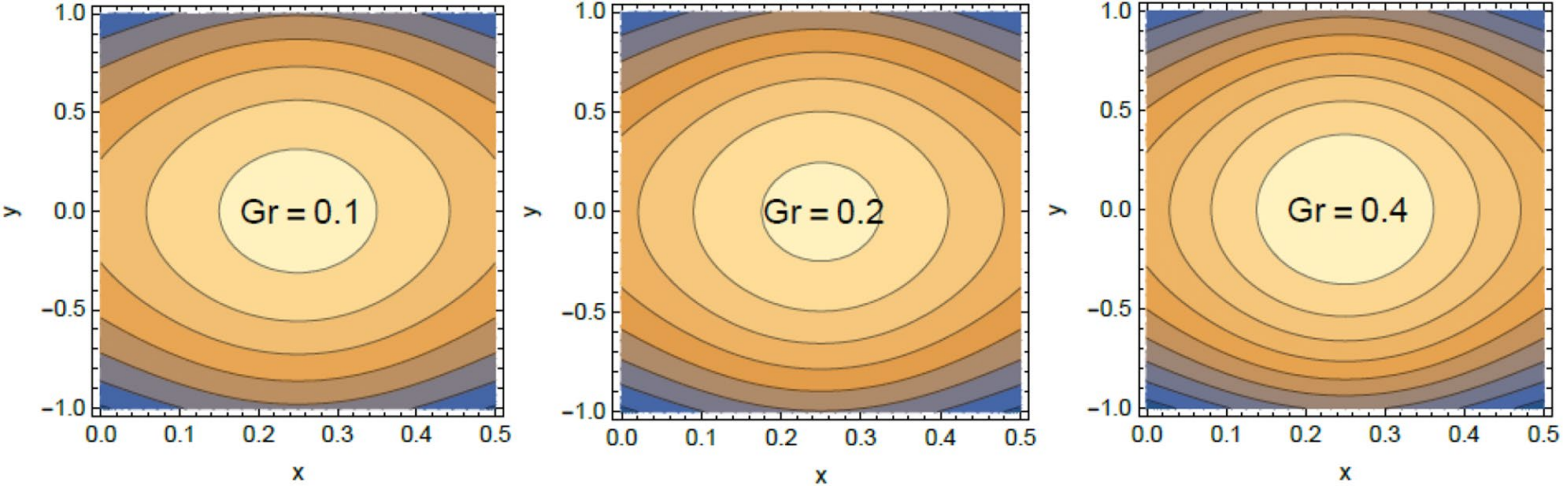
Contour plot of the speed for various amounts of the Grashof numeral $Gr$.

Accordingly, to explicate the current work physically, the influences of several factors are exemplified, and the outcomes are indicated in this section by a set of figures applying Mathematica software 12.0.0.0. The documented non-dimensional factors consist of the pseudoplastic parameter $\alpha$, the Grashof numeral $Gr$, Buoyancy ratio numeral $N_{r}$, Bioconvection Rayleigh numeral $R_{b}$, the Schmidt numeral $Sc$, the Prandtl numeral $\Pr$, the reaction rate factor $R_{d}$, the radiation coefficient $R$, the heat source coefficient $Q$, the Peclet numeral $Pe$, the bio-convection constant $\sigma$, and the thermophoretic parameter $\Gamma$. What follows highlights the impacts of these factors on speed, temperature, nano particle, and microorganism distributions. These mutual impacts will be exhibited through Figs. 2, 3, 4, 5, 6, 7, 8, 9, 10, 11, 12, 13, 14, 15, 16, 17, 18, and 19. The existing trapping phenomenon is also clarified across some Figs. 20, 21, 22 and 23 according to the variations of some of the above factors, and some evaluated amounts of skin friction are illustrated in Table 1.

**Table 1:** The skin friction parameter estimated at $y = h$ with the variations of $Gr$,$N_{r}$, $R_{b}$, and $\Pr$.

$Gr$	$N_{r}$	$\Pr$	$R_{b}$	$C_{f}$
0.1	0.2	0.7	0.3	0.357992
0.3	–	–	–	0.273975
0.5	–	–	–	0.189959
0.1	0.2	–	–	0.357992
–	0.4	–	–	0.376492
–	0.6	–	–	0.394992
–	0.2	0.1	–	0.348868
–	–	0.3	–	0.347538
–	–	0.5	–	0.346126
–	–	0.7	–	0.344633
–	–	–	0.1	0.344633
–	–	–	0.3	0.357992
–	–	–	0.5	0.371351

For further usefulness, the above-stated profiles are represented against the dimensionless variable $y$ for a few average amounts of the pertinent constraints, which change according to the debated factor in every diagram as:

$$\begin{gathered} \Pr = 0.7,\,\,Q = 0.5,\,\varepsilon = 0.2\,,\,\Gamma = 0.5,\,\,N_{r} = 0.3,\,\,R_{d} = 0.2,\,x = 0.5,\,Pe = 0.4,R_{b} = 0.3\,,\,R = 0.2,Gr = 0.2,\, \hfill \\ \sigma = 0.2,\,\alpha = 1,\,P = 0.4\,\,{\text{and}}\,\,\,Sc = 2. \hfill \\ \end{gathered}$$

### Skin friction coefficient

Table 1 contains certain estimations of the skin friction parameter at $y = h$ for different amounts of the Grashof numeral $Gr$, buoyancy ratio numeral $N_{r}$, bioconvection Rayleigh numeral $R_{b}$, and the Prandtl numeral $\Pr$. As seen from this table, it is clear that the rise of both $Gr$ and $\Pr$ reduces the skin friction factor, while the later rises sharply with the increase of the two buoyancy coefficients $N_{r}$ and $R_{b}$, which opposes the relations between the same parameters and the velocity field. These findings mean that the skin friction behaves opposite to the flow speed, which is logically understood.

### Velocity distribution

The velocity is the most important distribution that indicates the flow performance. Accordingly, velocity distribution $u$ is illustrated and expounded through Figs. 2, 3, 4, and 5 versus the non-dimensional variable $y$ for different associated parameters like the pseudo plastic parameter $\alpha$, the Grashof numeral $Gr$, buoyancy ratio numeral $N_{r}$, bioconvection Rayleigh numeral $R_{b}$. Figure 2 is schemed to display the behavior of $u$ under the impact of $\alpha$. It is found that $u$ improves with the rise of $\alpha$. In fact, this factor represents the non-Newtonian model involvement in the flow, so it is considered as a viscosity supply factor and as a result reduces the flow speed. This result accords with the same one^17^. Figure 3 signifies that speed increases with the growth of the Grashof coefficient $Gr$, which agrees with the results earlier obtained^41^. Actually, the Grashof numeral is a dimensionless quantity in a fluid movement with heat transmission, which distinguishes the fraction of the buoyancy force from the viscous force acting on the movement. This indicates that the viscosity of the fluid drops as $Gr$ rises, then the flow becomes easier, and the speed improves, as shown by Fig. 3. Figure 4 indicates that speed slightly increases near the limit $- h$, but after a very short distance it decays with the growth of the buoyancy ratio $N_{r}$. For more convenience, the buoyancy ratio is immediately proportionate to the concentration of nanoparticles scattered in the liquid. The rise of a set of these particles causes more resistance to the fluid flow and reduces its velocity. This result agrees with that first concluded^42^. The influence of bio convection Rayleigh numeral $R_{b}$ on the directional speed profile is explicated by Fig. 5. It is noticed that $R_{b}$ has the same impact as $N_{r}$, where it plays a dual role with the speed distribution, but the most common effect during flow is the decreasing effect of $R_{b}$ on the liquid speed. Actually, the bio-convection Rayleigh numeral characterizes the microorganisms concentration share in the buoyancy term, and the rise of these microorganisms density brings about a reduction in the liquid speed. This result approves with that concluded in^42^ and^43^.

### Heat distribution

The discussion of heat transfiguration is very important while analyzing the flows of liquids, particularly those associated by nanofluids. Figures 6, 7, 8, and 9 demonstrate the non-dimensional heat distribution $\theta$ against the non-dimensional parameter $y$ to understand the influences of the Prandtl numeral $\Pr$ in the cases of heat sink and source, the temperature resource stricture $Q$, and the radiation limitation $R_{d}$.

It is found that, the rise of the Prandtl numeral $\Pr$ reduces $\theta$ in the event of heat sink, as exposed in Fig. 6. By contrast, the increase of $\Pr$ enhances $\theta$ in the circumstance of heat source as shown by Fig. 7. Physically, Prandtl numeral signifies the ratio between the thickness of momentum diffusivity layer and thermal diffusivity layer, so it depends only on fluid properties. Subsequently, the growth of $\Pr$ corresponds to a low thermal conductivity and low tempertature diffusion, which is verified in the case of heat sink, as shown by Fig. 6. This outcome is consistent with that of^44^ and^45^. On the other hand, in the heat source case, it is obvious that the impact of the temperature source reflects the effect of $\Pr$ to be an enhancement factor of heat transport through fluid layers, as obtained by Fig. 7.

Figures 8 and 9 are designed to elucidate the influences of the temperature resource coefficient $Q$ and the radiation coefficient $R_{d}$ on the temperature exchange $\theta$. As concluded from these two figures, respectively, the rise of $Q$ enhances $\theta$, while the growth of $R_{d}$ reduces it. Logically, the rise of heat source leads to an enhancement in the heat distribution, as shown in Fig. 8, due to the plurality of heat basics. This finding is in accord with^42^ and^46^. Moreover, the radiation coefficient is considered substantially as a leakage factor of the internal heat. Therefore, as observed from Fig. 9, the rise of $R_{d}$, as an amount of radiating energy in all paths, leads to an outflow of temperature from hot bodies, which in turn cools the fluid and decreases heat. This outcome is in good concurrence as well as the outcomes achieved previously^47–50^.

### Nano-particles volume fraction profiles

This section is correlated to the impacts of the factors that affect the nanoparticles volume fraction $\phi$ and regulate its movement. Accordingly, Figs. 10, 11, 12, 13, 14, and 15 are schemed to demonstrate the developments in $\phi$ that are established by the presence and growth of the Schmidt numeral $Sc$, the heat source factor $Q$, the chemical reaction factor $R$, the radiation factor $R_{d}$, and the Prandtl numeral in the heat sink and source cases. It is shown that $\phi$ increases with the growth of both $Sc$ and $Q$, as seen from Figs. 10 and 11, whereas $\phi$ decays with the rise of $R$ and $R_{d}$, as attained by Figs. 12 and 13. Actually, the Schmidt numeral denotes the ratio of momentum to mass diffusivity, which is analogous to the Prandtl number in the mass transfer. Accordingly, mass diffusivity drops for larger amounts of $Sc$, which leads to an enhancement of $\phi$, as seen in Fig. 10. This finding agrees with the outcomes that were early obtained^51^ and^52^. Furthermore, the temperature resource is an item that produces or radiates heat; here this factor is found as an increasing factor of temperature which leads to more diffusion of nanoparticles through the flow and hence increases $\phi$, as realized by Fig. 11. On the contrary, as the chemical reaction coefficient $R$ increases, the mass concentration through the liquid decreases because the mass is not preserved in chemical reactions, where the important conservation law of the universe is the conservation of mass-energy. Higher amounts of $R$ lead to a drop in the chemical molecular diffusivity, i.e., less diffusion. Consequently, the concentration profile declines at all points of the movement field with the rise in the reaction factor, as obtained by Fig. 12. Moreover, as the heat radiation coefficient drops, the heat interchange and the nanoparticles condensation also decrease as a result. The last two outcomes are consistent with the early results attained^53^.

As shown in Figs. 14 and 15, the profile $\phi$ decays with the increase of the Prandtl numeral in the example of heat sink ($Q$ < 0) and rises with it in the situation of temperature resource ($Q$ > 0). Materially, the rise of $\Pr$ relates to a low thermal conductivity, which causes an enhancement in the nanoparticles concentration in the instance of temperature sink, Fig. 14. Nevertheless, it seems that the raise in the temperature due to the heat source (Fig. 15) reverses the effect of $\Pr$ to reduce the concentration of nanoparticles. These findings agree with the early findings achieved^54^.

### Microorganisms concentration distribution

Figures 16, 17, 18, and 19 are plotted to exhibit the distribution of microorganisms $\chi$ versus $y$ to clarify the impacts of the Peclet numeral $Pe$, the Schmidt numeral $Sc$, the bioconvection constant $\sigma$ and the chemical reaction coefficient $R$ on $\chi$. All these figures indicate that $\chi$ distribution drops with the increase of $Pe$, $Sc$, $\sigma$ and $R$. Significantly, $Pe$ represents the amount of the temperature delivery caused by fluid motion to temperature delivery resulting from thermal conductivity. Therefore, it is estimated that the increase of $Pe$ improves temperature transmission, which increases the spreading rate of microorganisms, constructing a lesser concentration of microorganism, as concluded from Fig. 16. This outcome is consistent with an early one^55^. In fact, the Schmidt numeral $Sc$ denotes the ratio of momentum to mass diffusivity, then the collective mass of microorganisms dissolves with the rise of $Sc$ and hence $\chi$ decreases, as seen from Fig. 17. This reduction effect of $Sc$ on the microorganism distribution $\chi$ agrees with that described earlier in^54^. Bio convection arrangements are usually experimented in the laboratory at low turbulences of randomly floating microorganisms which have a smaller density than the pure liquid. So, the rise of the bio convection constant $\sigma$ gives a drop in the concentration of these microorganisms, as displayed in Fig. 18. This result accords with the early results attained^54^. Similarly, the rise of the chemical reaction coefficient $R$ indicates a drop in the chemical molecular diffusivity, which indicates a reduction of the microorganism concentration $\chi$, as revealed in Fig. 19. This outcome is consistent with the early outcomes accomplished^56^.

### Trapping characteristic

**Figure 24:**
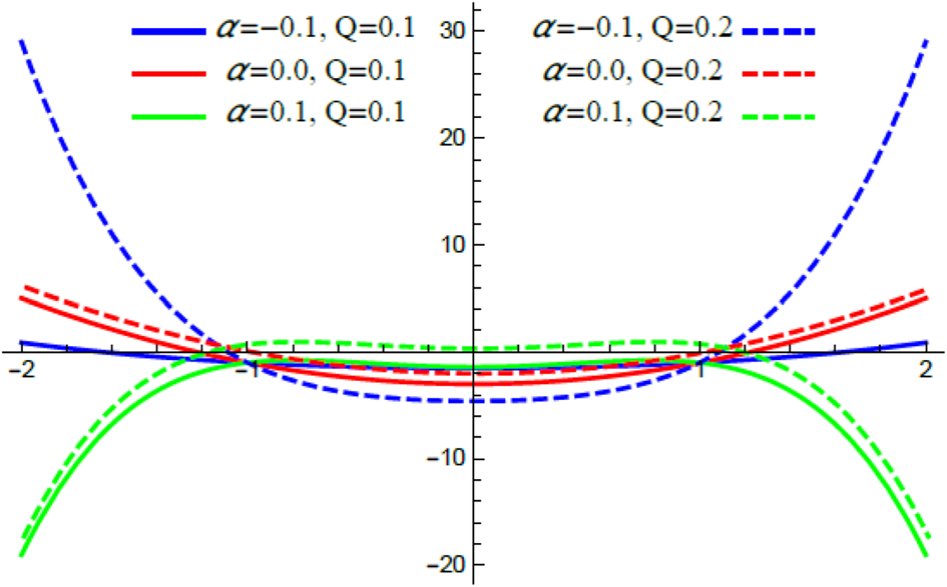
Velocity profile as appeared in Ref.^20^ in the case of $Gr = 0,\,R = 0,\,R_{d} = 0$.

Trapping is a significant physical phenomenon that occurs in the peristaltic flows. The streamlines distinguish the current pathways of liquid-particles flow, and the trapping characteristic signifies a creation of a curved bolus closed by streamlines or the contour of velocity. On the other side, under substantial restrictions, some of the streamlines divide and enclose a bolus. Normally, the profiles of streamlines and the edging wall in the wave frame are consistent, and the bolus transfers as a whole with the flow waves^57^. Figures 20, 21, 22, and 23 display the impacts of $R_{b}$, $N_{r}$, $Gr$ and $\alpha$ on the contour scheme for the axial velocity $u(x,y)$, where the radial direction $y$ is graphed versus the axial one $x$. It is found from Figs. 20, 21 and 22 that the volume of the trapped bolus decreases through the rise of $R_{b}$, $N_{r}$ and $\alpha$. Furthermore, Fig. 23 indicates that the scale of the trapped bolus rises with the rise of $Gr$, which are the same influences of these parameters on the speed vector distribution. These results are in consistence with the early results attained^20^. Figure 24 signifies the speed profile for various amounts of $\alpha$ and $Q$ in the special case $Gr = 0,\,R = 0,\,R_{d} = 0$ as obtained in Ref.^20^. It is noticed that this figure is approximately similar to Fig. 7a in^20^, the differences are only due to the different boundary conditions.

## Concluding remarks

The peristaltic MHD movement of an RF comprising microorganisms is considered in the present work. The flow takes place through a symmetric horizontal peristaltic channel. The current flow is shown under the influences of thermophoretic particles deposition, heat source, heat radiation and chemical reaction properties. The distinction of temperature, nanoparticles volume fraction, and microorganism concentration accompanying the velocity field are analyzed. The originality of the current study lies in the immersion of microorganisms like microbes or bacteria with speed, heat, and nano-particles distributions, examining the RF prototype as an appropriate non-Newtonian model. The model of the contemporary problem is believed to be relevant to many important applicable industrial, medical, and engineering manufacturing mechanisms. The liquids flow through the human body and the peristaltic flow pumps in several industrial and engineering machines are examples of the peristaltic flow style. Peristaltic pumps are optimistic transport pumps used widely in many engineering self-controls, for transmitting highly viscous fluids, or fluids with postponed solids. Additionally, the new feature of the current work comes from the necessity of understanding the damages or benefits that microbes, bacteria, and nanoparticles cause in the flow inside peristaltic tubes. It is considered important in the treatment of microbial intestinal diseases and the therapy of cancer. As usual, the assumptions of LWN and LRN are considered, and appropriate dimensional analysis is exemplified to convert a scheme of nonlinear partial differential equations that manage the numerous profiles of velocity, heat, nanoparticles volume fraction, and microorganism concentration to an ordinary system. The straightforward objective of all perturbation techniques is to simplify the nonlinear DEs by fragmenting the solution functions into many orders. The HPM is employed to comprehend semi analytical solutions. The pertinent non-dimensional physical parameters are caught, and a set of graphs are plotted to illustrate their characteristics. Moreover, assessments and validations of the theoretical deductions are carefully discussed. Additional studies on the present investigation can go in a variety of ways, such as considering diverse nano liquids, nanoparticle shapes, and the thermophysical characteristics of nanofluids. It is also anticipated that the current study would inspire experimental research into cooling nuclear reactors, high-heat plasmas, industrial managing machinery, and electronic elements to investigate more technological uses.

The major deductions are elicited from this research as follows:

- The effective parameters like the pseudo plastic parameter, buoyancy ratio numeral, bioconvection Rayleigh numeral are found to be coefficients that reduce the flow velocity, while the Grashof numeral improves it.
- The temperature diffusion rises in the situation of heat sink and decreases in that of temperature resource with the rise of the Prandtl numeral. Furthermore, the growth of the radiation coefficient reduces the heat transmission.
- The nano-particles volume fraction $\phi$ drops with the rise in the values of the reaction rate factor, the radiation coefficient, and the Prandtl numeral in the case of heat sink. On the other hand, $\phi$ increases with the growth of the Schmidt number, the temperature resource coefficient, and the Prandtl numeral in the instance of heat source.
- The microorganisms profile $\chi$ drops with the rise of all the operative parameters like the chemical reaction, the radiation, the Peclet, and the Schmidt coefficients. This means that the growth of all these factors aids in getting rid of the existing microorganisms, like harmful microbes, viruses, and bacteria in the human body tubes, such as the digestive system, large and small intestine.
- The streamlines are demonstrated, and the trapping behavior is discussed. It is found that the bolus size behaves like the speed profile for various parameters.
- A numerical table is included to illustrate the influences of the Grashof, buoyancy ratio, bioconvection Rayleigh, and the Prandtl numerals on the skin friction at the peristaltic wall.

## Supplementary Information

Supplementary Information.

## Data Availability

All data produced or analyzed throughout this research are contained in this manuscript.

## English symbols

$$T_{1} ,\,T_{2}$$: Walls temperatures
$$C_{1} ,\,\,C_{2}$$: Walls nanoparticles
$$N_{1} ,\,\,N_{2}$$: Walls microorganisms
$$t$$: Time
$$h$$: Wall model
$a$: Half channel width
$$b$$: Wave amplitude
$$c$$: Channel speed
$\underline{\underline{S}}$: Cauchy stress tensor
*U,V*: Fluid speed elements in $X\,{\text{and}}\,\,Y$ instructions, correspondingly
$$g$$: Gravity acceleration
$$p$$: Pressure
$$T$$: Liquid heat
$C$: Nanoparticle volume fraction
$$N$$: Microorganism concentration
*k*: Thermal conductivity
$$k^{*}$$: Thermophoretic coefficient
$Q_{1}$: Heat source/sink parameter
*D*_*B*_: Brownian diffusivity
$$V_{T}$$: Thermophoretic velocity
*R*_*1*_: Reaction rate constant
$$\overline{b}$$: Chemotaxis constant
$D_{m}$: Microorganism diffusion ratio
$W_{c}$: Maximum cell swaying velocity
$$(x,y)$$: Wave construction
$$C_{f}$$: Skin friction
$$u,\,v$$: $$x\,{\text{and}}\,\,y$$- Velocity components
$$Re\,$$: Reynolds numeral
$$Gr$$: Grashof numeral
$N_{r}$: Buoyancy ratio numeral
$R_{b}$: Bioconvection Rayleigh numeral
$$\Pr$$: Prandtl numeral
$R_{d}$: Thermal radiation parameter
$Q$: Heat source factor
$$Sc$$: Schmidt numeral
$R$: Chemical reaction factor
$$Pe$$: Peclet numeral
$$q$$: Synthetic parameter

## Greek symbols

$$\lambda$$: Wavelength
$\alpha^{*}$: Coefficient of pseudo-plasticity
$$\gamma^{.}$$: Shearing strain
$$\mu$$: Fluid viscosity
$$\rho_{f}$$: Fluid density
$\beta^{*}$: Volumetric expansion constant
$$\rho_{p}$$: Nanoparticles density
$$\rho_{m}$$: Microorganism density
$\gamma$: Average volume of microorganisms
(*ρC*)_*f*_: Heat capability of the liquid
(*ρC*)_*p*_: Heat capability of the nanoparticles
$\sigma^{*}$: Stefan Boltzmann constant
$$\delta$$: Wavelength parameter
$\alpha$: Pseudo plastic parameter
$$\theta$$: Dimensionless heat
$\phi$: Dimensionless nanoparticle volume fraction
$\chi$: Non-dimensional microorganisms
$$\Gamma$$: Thermophoretic parameter
$\sigma$: Bio-convection constant
$$\alpha$$: Pseudoplastic parameter
