# Supplementary material for: Peristaltic transport of Rabinowitsch nanofluid with moving microorganisms
Source: Sci Rep. 2023 Feb 1;13:1863. doi: 10.1038/s41598-023-28967-5 (PMC9892602; doi:10.1038/s41598-023-28967-5)
Supplement: Supplementary file 1 — Supplementary Information. [file 41598_2023_28967_MOESM1_ESM.pdf]

## Appendix

The following is a list of the constants that appear in Equations (42-44), (46) and (47):

$$a_1 = c_2 + c_6 + 1/2,\ a_2 = c_3 + c_7 + 1/2h,\ a_3 = c_4 + c_8,\ a_4 = c_5 + c_9,\ a_5 = c_{10},\ a_6 = c_{11},\ a_7 = c_{13} + c_{17} + 1/2,$$

$$a_8 = c_{14} + c_{18} + 1/2h,\ a_9 = c_{15} + c_{19},\ a_{10} = c_{16} + c_{20},\ a_{11} = c_{21},\ a_{12} = c_{22},\ a_{13} = c_{23} + c_{25} + 1/2,$$

$$a_{14} = c_{26} + 1/2h,\ a_{15} = c_{24} + c_{27},\ a_{16} = c_{28},\ a_{17} = c_{29},$$

$$\left.\begin{array}{l} a_{18} = -1 - c_{36}h^2/2 + c_{37}h^3/3 + c_{38}h^4/4 + c_{39}h^5/5 + c_{40}h^6/6 + c_{41}h^7/7 + c_{42}h^8/8 + c_{43}h^9/9 + c_{44}h^{10}/10 + \\ c_{45}h^{11}/11 + c_{46}h^{12}/12 + c_{47}h^{13}/13 + c_{48}h^{14}/14 + c_{49}h^{15}/15 + c_{50}h^{16}/16 + c_{51}h^{17}/17 + c_{52}h^{18}/18 + c_{53}h^{19}/19 \end{array}\right\},$$

$$a_{19} = c_{36}/2, a_{20} = -c_{37}/3, a_{21} = -c_{38}/4, a_{22} = -c_{39}/5, a_{23} = -c_{40}/6, a_{24} = -c_{41}/7, a_{25} = -c_{42}/8, a_{26} = -c_{43}/9, a_{27} = -c_{44}/10,$$

$$a_{28} = -c_{45}/11, a_{29} = -c_{46}/12, a_{30} = -c_{47}/13, a_{31} = -c_{48}/14, a_{32} = -c_{49}/15, a_{33} = -c_{50}/16, a_{34} = -c_{51}/17, a_{35} = -c_{52}/18,$$

and $a_{36} = -c_{53}/19$

where the constants $c_1, c_2, \ldots\ldots c_{53}$ can be printed as follows:

$$c_1 = Q\Pr/(1+R_d),\ c_2 = c_1h^2/4,\ c_3 = c_1h/12,\ c_4 = -c_1/4,\ c_5 = -c_1/12h,\ c_6 = c_1c_2h^2/2 + c_1c_4h^4/12,$$

$$c_7 = c_1c_3h^2/6 + c_1c_5h^4/20,\ c_8 = -c_1c_2/2,\ c_9 = -c_1c_3/6,\ c_{10} = -c_1c_4/12,\ c_{11} = -c_1c_5/20,\ c_{12} = \Gamma Sc,$$

$$c_{13} = c_{12}/8 + RSch^2/4,\ c_{14} = hRSc/12,\ c_{15} = -c_{12}/8h^2 - RSc/4,\ c_{16} = -RSc/12h,$$

$$c_{17} = -c_{12}c_{14}h/4 - c_{12}c_3h/4 + 7c_1c_{12}h^2/48 - c_{12}c_{16}h^3/8 - c_{12}c_5h^3/8 - c_{13}h^2RSc/2 - c_{15}h^4RSc/12,$$

$$c_{18} = c_{12}c_1h/12 - c_{12}c_{15}h/6 - c_4c_{12}h/6 - c_{14}h^2RSc/6 - c_{16}h^4RSc/20,$$

$$c_{19} = -c_{12}c_1/12 + c_{12}c_{14}/4h + c_3c_{12}/4h + c_{13}RSc/2,\ c_{20} = -c_{12}c_1/12h + c_{12}c_{15}/6h + c_4c_{12}/6h + c_{14}RSc/6,$$

$$c_{21} = -c_{12}c_1/48h^2 + c_{12}c_{16}/8h + c_5c_{12}/8h + c_{15}RSc/12,\ c_{22} = c_{16}RSc/20,$$

$$c_{25} = -c_{12}Pe/16 - c_{14}hPe/4 - c_{16}h^3Pe/8 - 7h^2PeRSc/48 - c_{12}Pe\sigma/8 - h^2PeRSc\sigma,$$

$$c_{26} = -c_{12}Pe/48h - c_{15}hPe/6 - c_{24}hPe/6 - hPeRSc/12 - hPeRSc\sigma,$$

$$c_{27} = c_{12}Pe/16h^2 + c_{14}Pe/4h + PeRSc/8 + c_{12}Pe\sigma/8h^2 + PeRSc\sigma/4,$$

$$c_{28} = c_{12}Pe/48h^3 + c_{15}Pe/6h + c_{24}Pe/6h + PeRSc\sigma/12h + PeRSc/12h,\ c_{29} = c_{16}Pe/8h + PeRSc/48h^2,$$

$$c_{30} = b_1 = -a_1Gr + a_7Gr\,N_r + P + a_{13}\,Gr\,R_b, c_{31} = b_2 = -a_2\,Gr/2 + a_8Gr\,N_r/2 + a_{14}Gr\,R_b/2,$$

$$c_{32} = b_3 = -a_3Gr/3 + a_9Gr\,N_r/3 + a_{15}Gr\,R_b/3, c_{33} = b_4 = -a_4Gr/4 + a_{10}Gr\,N_r/4 + a_{16}Gr\,R_b/4,$$

$$c_{34} = b_5 = -a_5\,Gr/5 + a_{11}Gr\,N_r/5 + a_{17}Gr\,R_b/5, c_{35} = b_6 = -a_6Gr/6 + a_{12}Gr\,N_r/6,$$

$$c_{36} = c_{30}, c_{37} = c_{31}, c_{38} = c_{32} + c_{30}{}^3\alpha, c_{39} = c_{33} + 3c_{30}{}^2\,c_{31}\alpha, c_{40} = c_{34} + 3c_{30}c_{31}{}^2\alpha + 3c_{30}{}^2c_{32}\alpha,$$

$$c_{41} = c_{35} + c_{31}{}^3\alpha + 6c_{30}\,c_{31}\,c_{32}\,\alpha + 3c_{30}{}^2c_{33}\alpha, c_{42} = 3c_{31}{}^2\,c_{32}\alpha + 3c_{30}\,c_{32}{}^2\alpha + 6c_{30}\,c_{31}\,c_{33}\alpha + 3c_{30}{}^2\,c_{34}\,\alpha,$$

$$c_{43} = 3\,c_{31}\,c_{32}{}^2\,\alpha + 3\,c_{31}{}^2\,c_{33}\alpha + 6\,c_{30}\,c_{32}\,c_{33}\alpha + 6\,c_{30}\,c_{31}\,c_{34}\alpha + 3\,c_{30}{}^2\,c_{35}\alpha,$$

$$c_{44} = c_{32}{}^3\alpha + 6\,c_{31}\,c_{32}\,c_{33}\alpha + 3\,c_{30}\,c_{32}{}^2\alpha + 3\,c_{31}{}^2\,c_{34}\alpha + 6\,c_{30}\,c_{32}\,c_{34}\alpha + 6\,c_{30}\,c_{31}\,c_{35}\alpha,$$

$$c_{45} = 3\,c_{32}{}^2\,c_{33}\,\alpha + 3\,c_{31}\,c_{33}{}^2\,\alpha + 6\,c_{31}\,c_{32}\,c_{34}\,\alpha + 6\,c_{30}\,c_{33}\,c_{34}\,\alpha + 3\,c_{31}{}^2\,c_{35}\,\alpha + 6\,c_{30}\,c_{32}\,c_{35}\alpha,$$

$$c_{46} = 3\,c_{32}\,c_{33}{}^2\,\alpha + 3\,c_{32}{}^2\,c_{34}\,\alpha + 6\,c_{31}\,c_{33}c_{34}\,\alpha + 3\,c_{30}\,c_{34}{}^2\,\alpha + 6\,c_{31}\,c_{32}c_{35}\,\alpha + 6\,c_{30}\,c_{33}\,c_{35}\alpha,$$

$$c_{47} = c_{33}{}^3\alpha + 6\,c_{32}\,c_{32}\,c_{34}\alpha + 3\,c_{31}\,c_{34}{}^2\alpha + 3\,c_{32}{}^2\,c_{35}\alpha + 6\,c_{31}\,c_{33}\,c_{35}\alpha + 6\,c_{30}\,c_{34}\,c_{35}\alpha,$$

$$c_{48} = 3\,c_{34}\,c_{33}{}^2\,\alpha + 3\,c_{34}{}^2\,c_{32}\alpha + 6\,c_{32}\,c_{33}\,c_{35}\alpha + 6\,c_{35}\,c_{31}\,c_{34}\alpha + 3\,c_{35}{}^2\,c_{30}\alpha,$$

$c_{49} = 3\,c_{34}{}^2\,c_{33}\alpha + 3\,c_{35}\,c_{33}{}^2\alpha + 6\,c_{32}\,c_{34}\,c_{35}\alpha + 3\,c_{35}{}^2\,c_{31}\,\alpha$, and

$$c_{50} = c_{34}{}^3\alpha + 6\,c_{33}\,c_{34}\,c_{35}\,\alpha + 3\,c_{35}{}^2c_{32}\alpha, c_{51} = 3\,c_{35}c_{34}{}^2\alpha + 3\,c_{35}{}^2c_{33}\alpha, c_{52} = 3\,c_{35}{}^2\,c_{34}\alpha, c_{53} = 3\,c_{35}{}^3\,\alpha.$$
